# Prenatal exposure to perfluoroalkyl sulfonic and carboxylic acids and neurodevelopmental delay in children up to 5 years old: Effect modification by progesterone and estradiol

**DOI:** 10.1016/j.eehl.2026.100238

**Published:** 2026-03-24

**Authors:** Haochen Lin, Qiong Zhang, Xiaona Chen, Yang Zhou, Guangzhen Liu, Yanying Wu, Feifei Qu, Longshen Fan, Dan Cai, Guanghui Dong, Shaoya Huang, Xiaowen Zeng

**Affiliations:** a Joint International Research Laboratory of Environment and Health, Ministry of Education, Guangdong Provincial Engineering Technology Research Center of Environmental Pollution and Health Risk Assessment, Department of Occupational and Environmental Health, School of Public Health, Sun Yat-sen University, Guangzhou 510080, China; b Maoming Maternal and Child Health Hospital, Maoming 525000, China; c Department of Public Health and Preventive Medicine, School of Medicine, Jinan University, Guangzhou 510632, China

**Keywords:** Per- and polyfluoroalkyl substances (PFAS), Perfluoroalkyl carboxylic acids (PFCAs), Perfluoroalkyl sulfonic acids (PFSAs), Progesterone, Estradiol, Neurodevelopmental delay

## Abstract

Per- and polyfluoroalkyl substances (PFAS) are persistent environmental pollutants associated with neurodevelopmental toxicity. However, the combined effects of PFAS subclasses and the modifying role of endogenous hormones remain poorly understood. Using data from the Maoming birth cohort (*n* = 543), we longitudinally assessed associations between prenatal exposure to perfluoroalkyl carboxylic acids (PFCAs) and perfluoroalkyl sulfonic acids (PFSAs) and developmental delay in children aged 3–60 months, employing generalized linear mixed models for individual PFAS and grouped weighted quantile sum regression for effects of PFAS mixtures. We further examined the effect modification by cord blood estradiol and progesterone, classified via latent profile analysis, and conducted molecular docking to explore potential receptor interactions. Both individual compounds [e.g., perfluorooctanoic acid and perfluorooctane sulfonic acid (PFOS)] and PFCA mixtures were associated with increased odds of developmental delay, particularly in total-ASQ, communication, and motor domains. These associations were significantly modified by latent profiles of cord blood hormones. For example, in the low-hormone profile, perfluorodecanoic acid was associated with fine motor delay (Odds ratio [OR] = 2.04; 95% CI: 1.18, 3.49), whereas linear-PFOS was associated with gross motor delay (OR = 4.76; 95% CI: 1.96, 11.54) in the high-hormone profile. Molecular docking indicated that most PFCAs have a preferential binding affinity for the ligand-binding domains of estradiol and progesterone receptors, supporting the biological plausibility of the observed hormonal effect modification. Our findings demonstrate that the association between prenatal PFAS exposure and neurodevelopment is subclass-specific and critically shaped by the in utero hormonal environment.

## Highlights

• A longitudinal study linked prenatal PFCAs/PFSAs to neurodevelopmental delay in children.
• PFCA mixtures exposure was associated with increased odds of total ASQ delay.
• Cord estradiol/progesterone levels modified prenatal PFCAs/PFSAs associations.
• Molecular docking showed preferential PFCA binding to ER/PR ligand-binding domains.

## Graphical abstract

**Figure:**
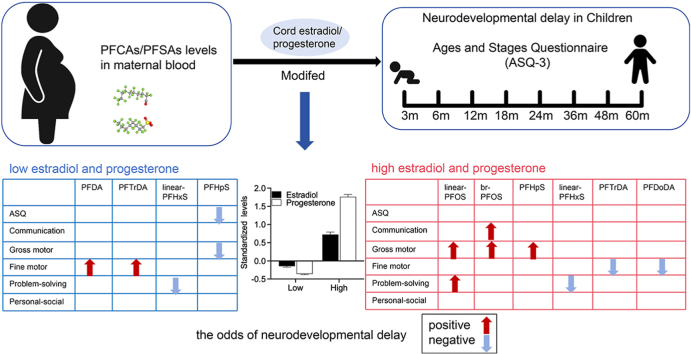

## 1 Introduction

Per- and polyfluoroalkyl substances (PFAS) represent a category of synthetic fluorinated chemicals employed in industrial and consumer applications owing to their chemical stability and surface-active properties [1,2]. These compounds are now ubiquitously detected in environmental matrices and human biological specimens, including those from pregnant women, prompting considerable public health apprehensions [[3], [4], [5]]. A large amount of data associates PFAS exposure with diverse adverse health outcomes [6], such as immunotoxicity, hepatotoxicity, reproductive dysfunction, and endocrine disruption. Specifically, the capacity of PFAS to traverse the placental barrier raises significant concerns regarding their impact on neurodevelopment processes during critical windows of fetal brain development [7,8]. Among PFAS subclasses, perfluoroalkyl sulfonic acids [PFSAs; e.g., perfluorooctane sulfonic acid (PFOS)] and perfluoroalkyl carboxylic acids [PFCAs; e.g., perfluorooctanoic acid (PFOA)] differ in structure and toxicokinetics [[9], [10], [11]], suggesting potentially distinct neurotoxic effects. Understanding these structure-activity relationships is critical for identifying the most hazardous PFAS compounds.

Animal and *in vitro* experiments have confirmed that PFAS could cross the blood-brain barrier, accumulate in the brain, and disrupt neurogenic processes, supporting biological plausibility for neurotoxicity [9,11]. However, epidemiological evidence on prenatal PFAS exposure and child neurodevelopment remains inconsistent [[12], [13], [14]]. So far, only two large cohorts have directly examined the link between prenatal PFAS exposure and developmental delay—a critical early marker of neurodevelopmental impairment [15,16]. One study in Japan found no associations between PFAS and developmental delays across JASQ-3 domains at age 4 [16], while another in China reported that prenatal PFOA and perfluorononanoic acid (PFNA) exposure was correlated with gross motor delay at 6 months [15]. Most previous studies also have employed cross-sectional designs or limited follow-up (≤48 months) [14,16,17], restricting insight into long-term outcomes.

Progesterone and estradiol, as neuroactive steroids, play essential regulatory roles in neural development processes, including synaptic plasticity, axonal growth [18], and myelin formation [19]. These hormones exhibit neuroprotective functions, such as enhancing mitochondrial activity and energy metabolism in adult rats [20], yet excessive prenatal progesterone has been associated with negative neurodevelopmental outcomes in offspring [21], and elevated estradiol levels have similarly been linked to neurotoxicity in zebrafish [22]. Previous studies have shown that PFAS may exhibit steroid-like or anti-sex hormone effects depending on the endogenous hormonal milieu [23,24]. *In vitro* studies further reveal that co-exposure to PFOS or PFOA and estradiol can amplify estradiol-mediated genomic and cellular responses [25], suggesting PFAS may modulate hormone signaling pathways in a context-dependent manner. Despite this, most epidemiological studies have treated sex steroid hormones as mediators rather than effect modifiers. Emerging evidence indicates that hormone levels modify PFAS-associated risks for preterm birth and hypertension [26,27], supporting their plausible role as modifiers in neurodevelopment. Given the important function of these hormones in fetal brain maturation and their interactions with PFAS, we hypothesize that cord blood estradiol and progesterone levels modify the association between prenatal PFAS exposure and childhood neurodevelopmental delay—a critical research gap this study aims to address.

Using a prospective birth cohort from Maoming, China, this study aims to: (1) systematically evaluate individual and mixture associations of PFAS subclasses (PFSAs/PFCAs) with neurodevelopmental delay in children from 3 to 60 months of age, and (2) assess effect modification by cord blood estradiol and progesterone, with preliminary mechanistic insights from *in silico* molecular docking.

## 2 Materials and methods

### 2.1 Study population

Participants were recruited from the Maoming Prospective Birth Cohort in China, with the study design and inclusion criteria established in prior research [17,28]. Of the 1030 initial mother-child pairs, 192 participants were excluded due to loss to follow-up, and 295 individuals lacked data on cord blood estradiol and progesterone measurements. In total, 543 mother-child pairs were included in this investigation (Fig. S1). The protocol (No. SYSU-2016-018) for this study received approval from the Human Research Committee at Sun Yat-sen University, and it was carried out in full conformity with the principles outlined in the Helsinki Declaration. Every pregnant woman who underwent an examination provided written informed consent.

### 2.2 PFAS measurement

Maternal serum samples were collected during the third trimester (average gestational age: 37.00 weeks) for the measurement of PFAS concentrations. Comprehensive methodologies for PFAS measurement have been described in other sources [29,30]. To quantify PFAS concentrations, we used ultra-performance liquid chromatography-tandem mass spectrometry (UPLC-MS/MS). Only 15 of 32 PFAS detected in maternal serum samples were used in the analysis. While we primarily included PFAS with a detection rate of more than 75% [31], branched perfluorohexanesulfonate (br-PFHxS, 53.22%) was also retained, given that branched and linear isomers may differ in effect [32]. These 15 compounds were further categorized into PFCAs and PFSAs based on their head groups [33]. The PFCAs included PFOA, perfluorobutanoic acid (PFBA), perfluoro-n-hexanoic acid (PFHxA), PFNA, perfluoro-n-decanoic acid (PFDA), perfluoro-n-dodecanoic acid (PFDoDA), perfluoro-n-undecanoic acid (PFUnDA), and perfluoro-n-tridecanoic acid (PFTrDA). The PFSAs included linear-PFHxS, linear-PFOS, br-PFOS, and perfluoro-1-heptanesulfonic acid (PFHpS). In this study, 6:2 and 8:2 chlorinated polyfluoroalkyl ether sulfonates (6:2 Cl-PFESA; 8:2 Cl-PFESA) were also classified within the PFSA subclass based on their shared sulfonic acid head group and biological persistence similar to PFOS [30,34]. Concentrations of PFAS beneath the limit of detection (LOD) were replaced by $\text{LOD}/\sqrt{2}$ [26], and LODs, abbreviations, and naming conventions are provided in Table S2.

### 2.3 Estradiol and progesterone measurements

During labor and delivery, we collected 5 mL of umbilical cord venous blood. Umbilical cord serum obtained by centrifugation was immediately stored at −80 °C. Serum estradiol and progesterone levels were quantified by chemiluminescence immunoassay utilizing an Agilent random-access assay system (Abbott Diagnostics, Abbott Park, USA). Specific experimental procedures have been described in previous literature [26,35]. Briefly, 50 μL of the umbilical cord serum samples were taken for the assay, and all samples were set up with duplicate wells for the assay. The final arithmetic mean of the results of the two assays was used as the hormone concentration of the sample. In this experiment, the LODs of progesterone and estradiol were 0.2 ng/mL and 5 pg/mL.

### 2.4 Neurodevelopmental assessment

In this study, we longitudinally assessed the neurodevelopmental status of 543 children, measured at 3, 6, 12, 18, 24, 36, 48, and 60 months of age. These measurements were performed by the children’s mothers utilizing the Ages & Stages Questionnaires, Third Edition (ASQ-3). This validated instrument is used for screening and monitoring children’s development and can assess children’s developmental skills in five key domains (communication, gross motor function, fine motor function, problem-solving ability, and personal-social skills) [36,37]. The questionnaire contains 30 distinct items that require maternal participants to indicate “yes”, “sometimes”, or “not yet” based on their observations of their child’s current capability to execute each specified task [38]. In this study, we recorded each child’s scores in the five domains and calculated total ASQ scores (higher scores indicate better levels of development). Each domain’s score should be between 0 and 60 [39]. Developmental delay was defined as a score more than two standard deviations (SDs) below the domain mean, according to the official criteria of the ASQ-3 and consistent with prior literature [39,40]. In addition, we considered delays in any domain of the ASQ-3 as a manifestation of overall developmental delay [41].

Neurodevelopmental assessments were administered under quiet conditions to ensure participant alertness and emotional stability, excluding children with fever, diarrhea, or infectious diseases [17].

### 2.5 Covariates

Potential confounders were identified beforehand based on a literature review [17,42,43] and a directed acyclic graph (DAG; Fig. S2). Data regarding maternal age, education level (≤high school, >high school), nutritional supplementation during pregnancy (yes or no), annual family income (less than 30,000, 30,000–100,000, or more than 100,000 Chinese Yuan), and breastfeeding duration (0, ≤6, or >6 months) were collected using standardized questionnaires administered by uniformly trained staff. These data were combined with prenatal physical examination records to obtain basic maternal characteristics, including body mass index before pregnancy (pre-BMI, kg/m^2^). Clinical information such as birth weight (g), parity (primipara/multipara), infant sex (male/female), and delivery method (vaginal/cesarean) was extracted from the hospital’s computer system of medical records, and a standardized process was used to ensure the quality of all data collection.

### 2.6 Statistical analysis

The distributions of maternal PFAS concentrations, umbilical cord serum progesterone and estradiol levels, child ASQ-3 scores, and other covariates were shown as mean ± standard deviation (SD), median [interquartile range (IQR)], or number (%), as appropriate. Demographic comparisons between the study cohort (*n* = 543) and excluded participants (*n* = 487) employed chi-square analyses for categorical variables and Wilcoxon rank-sum testing for continuous measures. PFAS concentrations were natural log-transformed (ln-PFAS) owing to their right-skewed distribution [44]. Spearman’s correlation analysis was performed to quantify pairwise correlations among PFAS. Missing values in covariates were managed using multiple imputation by chained equations (MICE) [45].

Restricted cubic spline regression (RCS) was used to evaluate potential nonlinear associations between individual ln-PFAS levels and developmental delay. The RCS model incorporated 3 knots positioned at the 10th, 50th, and 90th percentiles. This configuration was selected because it yielded the minimal Akaike Information Criterion (AIC) value. To account for repeated measures of child neurodevelopment, generalized linear mixed models (GLMM) were utilized to evaluate the associations between prenatal PFAS exposure and developmental delay in each of the five ASQ-3 domains, as well as overall developmental delay based on longitudinal ASQ data. The models included both the random intercept and random slope [2,46]. Additionally, the combined effects of the PFCAs/PFSAs mixture on the risk of developmental delay were assessed using grouped weighted quantile sum (GWQS) regression models. The GWQS method is tailored to handle mixtures of chemical groups, permitting the analysis of associations that may differ in both direction and magnitude. Previous studies have demonstrated that GWQS outperforms alternative methods in classification specificity, detection sensitivity, statistical power, and model fit [47,48]. We utilized the GWQS model for the binary outcome analysis, employing 10,000 bootstrap resamplings and adjusting for the identical confounders as in the logistic regression models.

To identify potential patterns of estradiol and progesterone during pregnancy, latent profile analysis (LPA) was used to fit a model for categories 1 to 5 based on z-standardized values of maternal estradiol and progesterone using the “gsem” command of STATA software. Based on the results of AIC, Bayesian information criterion (BIC), and the Lo-Mendell-Rubin test (Table S6) [49], we categorized pregnant women into two distinct profiles: Class 1 (low-estradiol and progesterone group, *n* = 451, 83.00%), characterized by below-average levels for both progesterone (*z* = −0.36) and estradiol (*z* = −0.15), and Class 2 (high-estradiol and progesterone group, *n* = 92, 17.00%), defined by significantly elevated levels of both hormones (progesterone *z* = 1.76; estradiol *z* = 0.73). Estradiol and progesterone profiles were defined by combining the results of fit indices. To assess potential effect modification, a PFAS × hormone profile interaction term was added to the GLMM. Wald tests were used to examine the significance of interaction effects.

Additionally, multiple sensitivity analyses were carried out to evaluate the robustness of the findings. First, additional potential confounders related to child neurodevelopment, including delivery method, birth weight, and breastfeeding duration, were incorporated into the GLMM and WQS models [17]. Second, the analyses were repeated in a complete-case dataset to test the impact of missing data imputation [43]. Third, generalized linear models were applied using continuous ASQ-3 scores to complement the main findings based on dichotomized developmental delay outcomes. Finally, inverse probability weighting (IPW) was applied within the GLMM, weighting included participants (*n* = 543) by their probability of inclusion to better represent the total population (*n* = 1030). All statistical analyses were conducted utilizing R (version 4.4.3) and STATA (version 17.0), with a two-sided significance threshold of 0.05.

### 2.7 Molecular docking analysis

We conducted molecular docking simulations to investigate the potential mechanisms underlying PFSA/PFCA toxicity. Using AutoDock Vina 2.1.6 software, docking structures [50,51] with optimal binding energy and high conformational stability were screened by setting the exhaustiveness parameter to 20 and using ERα (PDB No. 1GWR), ERβ (PDB No. 1QKM), and PR (PDB No. 1A28) as the target sites. PyMOL and Discovery Studio were combined to visualize the binding conformations [52,53].

## 3 Results

### 3.1 Characteristics of participants

In this study, we performed a detailed demographic characterization of 543 pregnant women and their newborns, and the results are shown in Table 1. The mean age of the pregnant women was 28.80 years, with an average pre-BMI of 21.16 kg/m^2^. Among the children, the mean gestational age was 37.00 ± 2.90 weeks, and the average birth weight was 2767 ± 630 g. Additionally, 45.12% of the children were girls. The mean concentrations of progesterone and estradiol in cord blood were 1274 ng/mL and 2831 pg/mL, respectively. The demographic traits of the study population and excluded participants were generally similar (Table S1). The results of repeated neuropsychological assessments are presented in Table S3.

**Table 1:** Demographic characteristics of the study population (*n* = 543).

Characteristics	Mean ± SD or n (%)
**Maternal age (years)**	28.80 ± 5.53
Missing	6 (1.10%)
**Pre-BMI (kg/m^2^)**	21.16 ± 4.79
Missing	59 (10.87%)
**Delivery method**	
Vaginal	308 (56.72%)
cesarean	231 (42.54%)
Missing	5 (0.74%)
**Parity**	
Primipara	307 (56.54%)
Multipara	232 (42.73%)
Missing	4 (0.73%)
**Gestational age (weeks)**	37.00 ± 2.90
Missing	6 (1.10%)
**Maternal education**	
≤high school	285 (52.49%)
>high school	212 (39.04%)
Missing	46 (8.47%)
**Family income (CNY/year)**	
<30,000	143 (26.34%)
30,000−100,000	209 (38.49%)
>100,000	88 (16.21%)
Missing	103 (18.97%)
**Nutrient supplementation during pregnancy**	
Yes	74 (13.63%)
No	412 (78.87%)
Missing	57 (7.50%)
**Infant sex**	
Male	294 (54.14%)
Female	245 (45.12%)
Missing	4 (0.74%)
**Birth weight (g)**	2767 ± 630
Missing	4 (0.74%)
**Breastfeeding duration**	
0 month	284 (52.30%)
≥6 months	123 (22.65%)
<6 months	76 (14.00%)
Missing	60 (11.05%)
**Progesterone (ng/mL)**	1274 ± 717
**Estradiol (pg/ml)**	2831 ± 1796

For continuous variables, use mean (SD); for categorical variables, use *n* (%). Pre-BMI, body mass index before pregnancy; CNY, Chinese Yuan.

### 3.2 Concentrations of PFAS

Among the 15 PFAS analyzed, all were detected in over half of the samples, with detection frequencies ranging from 53.22% to 100.00%. The most frequently detected compounds were br-PFOS (100.00%), followed by PFNA, PFDA, linear-PFOS, and 6:2 Cl-PFESA (all 99.82%), and PFOA (99.45%). The median total concentrations of PFCAs and PFSAs were 4.01 ng/mL (IQR: 2.89–5.70 ng/mL) and 5.55 ng/mL (IQR: 3.74–9.03 ng/mL), respectively. Among PFCAs, the highest median concentrations were observed for PFOA (1.05 ng/mL, IQR: 0.75–1.47 ng/mL) and PFBA (0.85 ng/mL, IQR: 0.12–1.77 ng/mL). For PFSAs, the median (IQR) concentrations of linear-PFOS, br-PFOS, and 6:2 Cl-PFESA were 3.64 (2.45–5.88), 0.85 (0.51–1.41), and 0.68 (0.43–1.09) ng/mL, respectively. Detailed distributions of PFAS are presented in Table 2.

**Table 2:** Detection rates and concentration distributions of perfluoroalkyl substances in maternal serum (ng/mL; *n* = 543).

PFAS	25th percentile	Median	75th percentile	Detection rate (%)
**PFCAs**				
PFBA	0.12	0.85	1.77	78.08%
PFHxA	0.01	0.02	0.07	79.37%
PFOA	0.75	1.05	1.47	99.45%
PFNA	0.32	0.44	0.59	99.82%
PFDA	0.27	0.38	0.53	99.82%
PFUnDA	0.37	0.54	0.74	99.26%
PFDoDA	0.01	0.04	0.07	77.16%
PFTrDA	0.22	0.36	0.54	99.26%
ΣPFCAs a	2.89	4.01	5.70	—
**PFSAs**				
linear-PFHxS	0.10	0.16	0.26	98.90%
br-PFHxS	0.001	0.002	0.004	53.22%
6:2 Cl-PFESA	0.43	0.68	1.09	99.82%
linear-PFOS	2.45	3.64	5.88	99.82%
br-PFOS	0.51	0.85	1.41	100.00%
PFHpS	0.02	0.05	0.09	85.45%
8:2 Cl-PFESA	0.003	0.01	0.02	78.08%
ΣPFSAs b	3.74	5.55	9.03	—

Table S2 lists the full names and abbreviations for all PFAS analyzed in this study.

a Sum of PFBA, PFHxA, PFOA, PFNA, PFDA, PFUnDA, PFDoDA, and PFTrDA.

b Sum of linear-PFHxS, br-PFHxS, 6:2 Cl-PFESA, linear-PFOS, br-PFOS, PFHpS, and 8:2 Cl-PFESA.

### 3.3 Association between PFCAs/PFSAs and developmental delay

On average, each 1-log-unit increase in PFAS was linked to higher odds of developmental delay. Specifically, PFOA was associated with increased odds of total developmental delay (Odds Ratio [OR] = 1.32; 95% CI: 1.05, 1.64). In domain-specific analyses, linear-PFOS and PFDA were correlated with higher odds of delay in fine motor function. Additionally, PFOA and br-PFOS were linked to higher odds of impaired problem-solving ability (PFOA: OR = 1.21, 95% CI: 1.20, 1.22; br-PFOS: OR = 1.11, 95% CI: 1.10, 1.12). For personal-social skills, positive associations were observed for PFDA, PFUnDA and 6:2 Cl-PFESA, adjusted for covariates (Table 3). Conversely, PFHxA, PFNA, br-PFHxS, 8:2 Cl-PFESA, PFBA, and PFDoDA were associated with reduced odds of developmental delay in specific domains. The crude models also showed comparable results (Table S4). However, nonlinear associations were observed for PFHxA, PFOA, and PFDoDA with total ASQ, while no significant nonlinear trends were found for the other PFAS (Fig. S4).

**Table 3:** Adjusted ORs (95% CIs) per unit increase in prenatal PFAS levels for developmental delay and grouped weighted quantile sum (GWQS) regression models in children aged 3–60 months: A longitudinal analysis (*n* = 543).

Serum PFAS (ng/mL)	ASQ	Communication	Gross motor function	Fine motor function	Problem-solving ability	Personal-social skills
	Adjusted ORs (95% CIs)					
**PFCAs**						
PFBA	1.032 (0.959, 1.109)	1.152 (0.995, 1.335)	1.039 (0.906, 1.192)	0.975 (0.868, 1.096)	**0.897 (0.891, 0.903)**	0.970 (0.791, 1.189)
PFHxA	0.995 (0.890, 1.113)	1.141 (0.916, 1.420)	0.966 (0.785, 1.190)	0.906 (0.751, 1.092)	**0.688 (0.510, 0.928)**	0.940 (0.511, 1.730)
PFOA	**1.315 (1.052, 1.644)**	1.376 (0.920, 2.059)	1.177 (0.800, 1.732)	1.149 (0.793, 1.666)	**1.209 (1.204, 1.215)**	1.566 (0.791, 3.103)
PFNA	1.186 (0.852, 1.650)	1.175 (0.599, 2.302)	0.980 (0.542, 1.773)	1.356 (0.749, 2.453)	0.718 (0.357, 1.446)	**0.965 (0.963, 0.967)**
PFDA	1.281 (0.968, 1.694)	1.171 (0.679, 2.020)	0.996 (0.597, 1.661)	**1.538 (1.534, 1.541)**	1.514 (0.532, 4.303)	**1.251 (1.242, 1.260)**
PFUnDA	1.232 (0.942, 1.610)	1.275 (0.761, 2.137)	1.093 (0.677, 1.762)	1.245 (0.783, 1.978)	1.151 (0.449, 2.952)	**1.159 (1.153, 1.164)**
PFDoDA	0.918 (0.802, 1.051)	0.934 (0.714, 1.222)	**0.786 (0.620, 0.996)**	0.988 (0.778, 1.254)	**0.671 (0.670, 0.673)**	0.826 (0.622, 1.097)
PFTrDA	0.946 (0.758, 1.181)	0.860 (0.585, 1.263)	0.845 (0.562, 1.268)	0.967 (0.672, 1.391)	1.075 (0.456, 2.534)	1.042 (0.535, 2.028)
GWQS index1	**1.360 (1.100, 1.681)**	**1.625 (1.104, 2.393)**	1.384 (0.891, 2.149)	1.364 (0.899, 2.070)	**0.993 (0.987, 0.998)**	1.355 (0.699, 2.627)
**PFSAs**						
linear-PFHxS	1.189 (0.992, 1.425)	1.121 (0.808, 1.555)	1.020 (0.733, 1.420)	1.253 (0.915, 1.717)	1.006 (0.547, 1.849)	1.280 (0.772, 2.122)
br-PFHxS	1.055 (0.924, 1.204)	0.993 (0.769, 1.283)	**0.858 (0.857, 0.860)**	0.856 (0.679, 1.080)	1.022 (0.784, 1.333)	1.036 (0.818, 1.312)
6:2 Cl-PFESA	1.193 (0.943, 1.508)	1.544 (0.978, 2.437)	1.328 (0.856, 2.058)	1.247 (0.829, 1.877)	1.081 (0.470, 2.485)	**1.156 (1.149, 1.164)**
linear-PFOS	1.147 (0.933, 1.411)	1.517 (0.984, 2.340)	1.126 (0.772, 1.642)	**1.284 (1.281, 1.288)**	0.854 (0.421, 1.732)	1.219 (0.625, 2.379)
br-PFOS	1.114 (0.899, 1.382)	1.354 (0.919, 1.995)	1.052 (0.707, 1.567)	1.076 (0.752, 1.539)	**1.111 (1.104, 1.118)**	1.070 (0.536, 2.134)
PFHpS	0.934 (0.839, 1.040)	0.981 (0.791, 1.217)	0.958 (0.781, 1.176)	0.952 (0.804, 1.126)	0.709 (0.492, 1.021)	0.839 (0.620, 1.136)
8:2 Cl-PFESA	1.011 (0.876, 1.166)	1.100 (0.827, 1.463)	0.992 (0.759, 1.296)	0.995 (0.763, 1.299)	1.042 (0.761, 1.427)	**0.798 (0.792, 0.805)**
GWQS index2	1.063 (0.851, 1.327)	1.211 (0.833, 1.760)	1.148 (0.844, 1.562)	1.251 (0.917, 1.706)	0.891 (0.174, 4.566)	**1.161 (1.158, 1.163)**

GWQS index 1 uses all PFCA types, and GWQS index 2 uses all PFSA types to reflect their mixed exposure levels. Odds ratios and 95% confidence intervals are reported to three decimal places due to numerically close confidence interval bounds. Values in bold represent *P* < 0.05. Models were adjusted for maternal age, infant sex, family income, pre-BMI, maternal education, parity, and nutrient supplementation during pregnancy.

**Fig. 1:**
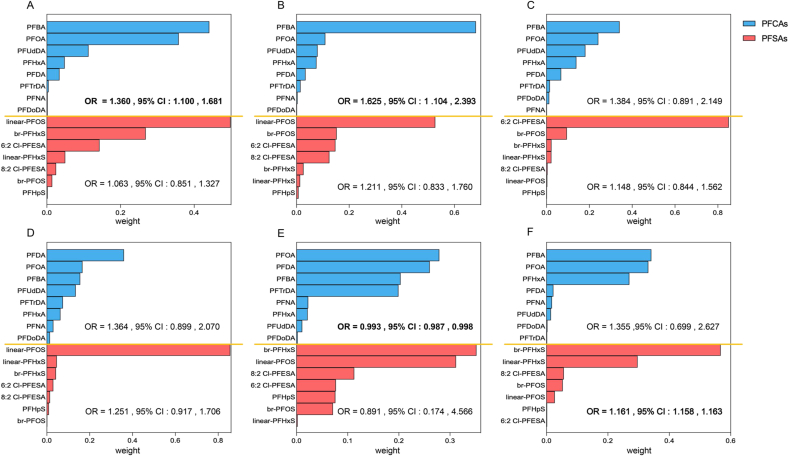
The weights of individual PFAS within the PFCAs and PFSAs groups were estimated using grouped weighted quantile sum (GWQS) regression models for six neurodevelopmental domains: (A) ASQ, (B) Communication, (C) Gross motor function, (D) Fine motor function, (E) Problem-solving ability, and (F) Personal-social skills. Values in bold represent *P* < 0.05. Models were adjusted for maternal age, infant sex, family income, pre-BMI, maternal education, parity, and nutrient supplementation during pregnancy.

GWQS regression indicated complex associations of the PFCA mixture with developmental delay. For instance, each quartile increase in the PFCA mixture was correlated with 36% higher odds of developmental delay based on the total ASQ assessment (OR = 1.36; 95% CI: 1.10, 1.68), and 63% higher odds of communication delay (OR = 1.63; 95% CI: 1.10, 2.39). PFBA contributed the most to the mixture effect in both models (weights: 0.44 for total ASQ, 0.68 for communication). Conversely, the mixture was associated with reduced odds of problem-solving delay (Fig. 1 and Table S5). For PFSAs, the mixture was positively linked with the odds of delay in personal-social skills, with br-PFHxS showing the highest weights (0.57).

### 3.4 Interaction of PFCAs/PFSAs with estradiol and progesterone in developmental delay

**Fig. 2:**
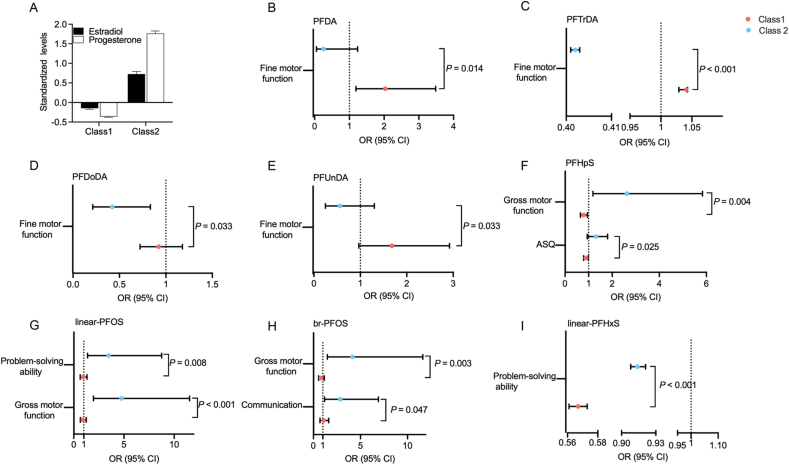
Interaction of PFAS exposure with estradiol/progesterone levels on neurodevelopmental delay. (A) Latent Profile Analysis (LPA) categorized the sample into two groups based on *z*-score standardized progesterone and estradiol levels: Class 1 (low estradiol and progesterone, 83.00%) characterized by below-average levels of progesterone (*z* = −0.36) and estradiol (*z* = −0.15), and Class 2 (high estradiol and progesterone, 17.00%) characterized by elevated levels of progesterone (*z* = 1.76) and estradiol (*z* = 0.73). (B-I) Associations of prenatal exposure to PFCAs and PFSAs with neurodevelopmental delay, assessed using generalized linear mixed models (GLMM) and stratified by latent profiles of cord blood estradiol and progesterone. Models were adjusted for maternal age, infant sex, family income, pre-BMI, maternal education, parity, and nutrient supplementation during pregnancy. *P*-values represent interaction tests between PFCAs/PFSAs exposure and estradiol/progesterone levels. More details of the association are presented in Table S7.

To evaluate the interaction effects of cord progesterone and estradiol, we used LPA to classify participants into two distinct hormonal profiles: Class 1 (low estradiol and progesterone) and Class 2 (high estradiol and progesterone). Overall, we observed statistically significant interactions between the latent hormone profiles and prenatal PFCAs/PFSAs regarding developmental delay (Fig. 2, Table S7).

For PFCAs, the estimated ORs tended to be higher among participants with low estradiol and progesterone levels than among those with high hormone levels. In particular, in the low-estradiol and progesterone group, PFDA was correlated with increased odds of fine motor delay (OR = 2.04; 95% CI: 1.19, 3.49). In contrast, in the high-estradiol and progesterone group, PFDoDA was associated with reduced odds of fine motor delay (OR = 0.42; 95% CI: 0.21, 0.83). PFTrDA exhibited associations in both profiles but in opposite directions, showing increased odds in the low-hormone group and reduced odds in the high-hormone group. PFUnDA exhibited a similar trend, with a higher OR in the low-estradiol and progesterone group than in the high-hormone group, yet these associations did not reach statistical significance (Fig. 2E).

Conversely, PFSAs followed an opposite pattern, with estimated ORs being consistently higher in the high-estradiol and progesterone group than in the low-hormone group. With regard to gross motor function, PFHpS, linear-PFOS, and br-PFOS were significantly related to higher odds of delay in this high-hormone group (OR_PFHpS_ = 2.63, 95% CI: 1.18, 5.83; OR_linear-PFOS_ = 4.76, 95% CI: 1.96, 11.54; and OR_br-PFOS_ = 4.15, 95% CI: 1.48, 11.63). For communication and problem-solving, elevated odds of delay were also observed for br-PFOS and linear-PFOS, respectively. In contrast, in the low-estradiol and progesterone group, PFHpS and linear-PFHxS were correlated with reduced odds of developmental delay. Linear-PFHxS was linked to reduced odds of developmental delay in both profiles, with a more pronounced reduction in the low-estradiol and progesterone group.

### 3.5 Molecular docking analysis of different structural types of PFCAs/PFSAs with estrogen and progesterone receptors

We performed exploratory molecular docking for several PFAS that exhibited significant interactions in the epidemiologic analysis (e.g., PFDA, PFDoDA, PFTrDA, PFUnDA, PFOS, PFHxS) against ERα, ERβ, and PR (Figs. S5–S7). Overall, predicted binding energies ranged from −10.6 to −6.4 kcal/mol. Notably, several PFCAs displayed preferential binding affinities for ERα and PR (e.g., PFDoDA–PR = −10.6 kcal/mol; PFDA–PR = −10.0 kcal/mol; PFUnDA–ERα = −9.8 kcal/mol) and formed short-range hydrogen bonds or polar contacts with conserved residues (e.g., ARG, TRP, LYS, GLN) in the binding pocket.

### 3.6 Sensitivity analysis

Sensitivity analyses validated the robustness of the main findings. Adjustment for additional covariates, including delivery method, birth weight, and breastfeeding duration, yielded similar effect estimates (Table S8). Analyses restricted to participants with complete data produced consistent results (Table S9). Cross-sectional generalized linear models also showed patterns in line with the longitudinal findings, though effect estimates varied by exposure and developmental domain (Tables S10–S17). Finally, we employed IPW to evaluate the correlation between PFAS exposure and developmental delay, with the majority of results remaining substantially consistent (Table S18).

## 4 Discussion

Our longitudinal findings indicate that prenatal exposure to specific PFCAs/PFSAs is associated with elevated odds of developmental delay across key neurodevelopmental domains from infancy through preschool age. Notably, these associations demonstrate a striking hormone-dependent pattern. PFSAs (particularly PFHpS and PFOS) exhibit stronger neurodevelopmental toxicity in high estradiol and progesterone conditions, while PFCAs (including PFDA and PFTrDA) show greater adverse effects under low estradiol and progesterone conditions. These findings offer novel insights into potential mechanisms underlying the neurodevelopmental impacts of PFCAs/PFSAs exposure during early childhood.

### 4.1 Longitudinal association between individual and mixed PFCAs/PFSAs exposure and developmental delay in children

PFOS and PFOA were commonly detectable in our cohort, with median concentrations of linear PFOS and PFOA of 3.64 and 1.05 ng/mL, respectively. These medians are typically inferior to those documented in several preceding birth cohorts [54,55]. Previous studies suggest prenatal PFAS concentrations have declined over time [56,57], so differences between our results and earlier reports may reflect different sampling periods. Notably, despite these comparatively lower exposure levels, our analyses revealed significant associations with child neurodevelopmental outcomes.

Specifically, our study provides further evidence that prenatal exposure to certain PFAS relates to heightened odds of developmental delay in early childhood. In single-pollutant models, higher maternal serum levels of 6:2 Cl-PFESA, PFOA, PFOS, PFDA, and PFUnDA were significantly correlated with elevated odds of developmental delay—particularly in the fine motor, problem-solving, and personal-social domains. These results align with prior cohorts associating PFAS with neurodevelopmental deficits [17,58], although other studies have reported null or protective associations [16], likely reflecting heterogeneity in study designs and outcome definitions [59,60].

However, while numerous studies have examined the association between PFAS and continuous neurodevelopmental scores [59,61,62], the relationship with developmental delay—as identified by standardized screening tools [40]—remains poorly characterized. Shifting the focus from subtle population-level shifts in mean scores to the identification of children at the lower end of the developmental distribution holds significant public health relevance, as such children represent a high-priority subgroup for targeted monitoring and intervention to align with preventive public health goals. So far, only two large birth cohorts have directly assessed the association between prenatal PFAS exposure and developmental delay defined by standardized screening tools. For instance, data from the Japan Environment and Children’s Study did not observe significant links between developmental delay at age 4 and eight PFAS congeners [16], whereas the Wuhu Birth Cohort in China reported trimester-specific effects of PFOA and PFNA on gross motor delay at 6 months [15]. Compared with these existing studies, our research provides further insight into long-term dynamics by extending the follow-up period to 60 months.

Leveraging a longitudinal design with repeated measures from 3 to 60 months, we were able to assess the association between PFAS exposure and developmental delay dynamics more reliably than previous cross-sectional studies [15]. A key advance is our use of a binary developmental delay outcome—defined by validated, official ASQ-3 cut-offs (≥2SDs below the mean for any domain) [[63], [64], [65]]—which moves beyond mean score differences to identify children at high risk of clinically meaningful delay. This approach aligns with screening methodologies used in prior studies [66,67] and addresses the gap in research on standardized, screening-defined delay. When combined with GLMM for robust handling of repeated measures and large sample sizes, ensuring high statistical power, our methodology offers strong and nuanced evidence linking prenatal PFAS exposure to increased odds of early developmental delay.

In contrast to the positive associations, inverse associations were also observed for several PFAS (e.g., PFHxA and PFDoDA) in specific domains. These findings should be interpreted with caution. One plausible explanation involves the non-monotonic dose–response behavior often reported for endocrine-disrupting chemicals [68], supported by our RCS analyses that indicated significant nonlinearity for PFHxA and PFDoDA with total ASQ (Fig. S4). Such biphasic patterns, where effects reverse across exposure levels, are plausible for PFAS; for example, zebrafish studies have shown higher locomotor activity at 0.4 μM than at 1.0 μM PFOS, a clear NMDR effect [69]. Additionally, these inverse associations may arise from unmeasured confounding due to complex co-exposures. For example, fish and shellfish consumption may increase PFAS body burdens, while also providing neurobeneficial nutrients [70]. In this setting, a higher level of a specific PFAS may serve as an indicator of a broader dietary pattern or mixture of exposures, rather than its isolated effect.

To dissect the joint neurodevelopmental impacts of PFAS co-exposure, we employed GWQS regression, a method uniquely suited to handle mixtures with divergent association directions by incorporating group-level weights, exhibiting better goodness-of-fit, sensitivity, and statistical power relative to conventional methods [47,48]. Our analysis further revealed structure-dependent neurotoxicity, with PFCAs mixtures showing stronger associations with total ASQ and communication deficits, while PFSAs mixtures were associated only with poorer personal-social skills, mainly driven by PFBA, PFOA, and br-PFHxS. This divergence underscores the importance of considering chemical subclass in risk assessments, likely reflecting differences in bioaccumulation and biological activity [71,72]. While most previous studies focused on individual PFAS, limited research has assessed mixture effects on early neurodevelopment. A case-control study (*n* = 551) found that prenatal exposure to PFAS mixtures (mainly PFOA, PFHxS, PFHpA, PFOS, and PFNA) was linked to increased ASD risk (OR = 1.57; 95% CI: 1.16, 2.13), suggesting potential neurodevelopmental risks from gestational PFAS co-exposure [73]. Similarly, the Shanghai Birth Cohort Study showed that PFAS mixture (mainly PFOA, PFHxS, PFUnDA, and PFOS) was significantly correlated with reductions in cognitive and language scores [14]. These findings are consistent with ours and highlight the neurotoxic potential of PFAS co-exposure [74]. However, our study advances this field by identifying PFBA as a key driver of PFCA mixture toxicity—a novel insight potentially attributable to its high bioavailability and estrogen-related receptor gamma (ERRγ)-mediated activity [75,76]. Moreover, by integrating mixture modeling with longitudinal analysis and subclass differentiation, we provide a more realistic and mechanistically nuanced perspective on the developmental neurotoxicity of PFAS, highlighting the value of structure-based mixture assessment in future environmental health studies.

### 4.2 Associations between prenatal PFCAs/PFSAs exposure and developmental delay: Modification by estradiol and progesterone

Cord blood estradiol and progesterone are commonly used biomarkers of the late-gestation fetal endocrine milieu [77]. Experimental studies have verified that progesterone and estradiol provide neuroprotection by regulating mitochondrial function. These hormones promote the upregulation of energy metabolic pathways and enhance antioxidant defenses, both important for preserving neuronal integrity [20]. Nevertheless, data from epidemiological cohort studies have illuminated the potential long-term detrimental effects of prenatal progesterone exposure on the neurodevelopment of offspring [21]. Likewise, estrogen exposure has demonstrated neurotoxic effects in zebrafish models [22]. In the absence of hormones, PFAS mimic steroids. When endogenous hormones are present, PFAS bind to different sites on receptors, disrupting the biological activity of hormones and leading to anti-sex hormone effects [23,24]. Mechanistic studies demonstrate that simultaneous exposure to PFOS or PFOA and estradiol can enhance estrogenic signaling [25], which may in turn impair neuronal function [22]. Additionally, a recent Chinese cohort study indicated that estrogen levels modify the association between PFAS exposure and preterm birth, suggesting a modulating role of sex steroids in PFAS toxicity [26]. Other research has underscored potential antagonistic interactions between PFAS and sex steroids, which may influence susceptibility to outcomes such as hypertension [27]. These findings reinforce the plausibility of sex steroids serving as effect modifiers for PFAS toxicity. To our knowledge, no prior research has explored the role of estradiol and progesterone in modifying the association between PFAS exposure and developmental delay, highlighting the novelty of our findings and the necessity for additional validation.

Using LPA, we characterized their joint distribution as latent endocrine profiles, providing a more integrated representation of fetal endocrine status than modeling either hormone alone and enabling the identification of subgroups with differential susceptibility to PFAS exposure [78]. In our data, we observed subclass-specific effect modification, generally characterized by stronger adverse associations for PFCAs at lower hormone levels and for PFSAs at higher levels.

Our molecular docking analyses suggested that multiple PFAS congeners detected in the cohort (including representative PFCAs and PFSAs) could adopt energetically stable conformations within the ligand-binding domains of ERα/ERβ and PR, providing molecular-level insights into the observed hormonal modification. Although computational predictions are preliminary, prior studies combining *in silico* modeling with *in vitro* bioassays have reported correlations between PFAS–estrogen receptor binding and biological potency [24,79]. The majority of PFCAs exhibit stronger binding affinities to ERα and PR (e.g., PFDoDA–PR: −10.6 kcal mol^−1^), forming stable interactions with key residues (e.g., ARG, GLN), consistent with *in vitro* studies characterizing them as “weak xenoestrogens” [80]. Since PFCAs demonstrate a much weaker affinity for estrogen receptors compared to native hormones, elevated physiological hormone levels presumably prevent PFCAs from occupying these receptors [80]. Conversely, in the low-hormone profile, reduced competition allows PFCAs to interact more readily with receptors, facilitating their receptor-mediated toxicity. PFSAs such as PFOS, however, showed weaker binding modes, potentially relying more on non-receptor pathways such as the induction of oxidative stress [81]. Experimental results suggested that elevated ER expression may amplify PFOS toxicity, indicating this as a potential underlying mechanism for our results [82,83]. This hormone-dependent toxic difference may also be closely associated with the chemical structural characteristics of PFAS. PFCAs containing carboxyl groups exhibit relatively weak bioaccumulation potential, while PFSAs with sulfonic acid groups display stronger binding affinity to tissue proteins [84]. Such structural divergence may further influence their binding affinity to hormone receptors or the activation of non-receptor-mediated pathways under varying hormone levels. Further experimental validation is warranted to substantiate these structure-activity relationships and elucidate the mechanisms underlying the observed differential toxicity.

Taken together, these mechanistic insights confer biological plausibility to the observed associations between PFAS exposure, hormonal status, and neurodevelopment. They underscore the complexities of chemical structure, endocrine regulation, and neurobehavioral outcomes, reinforcing the need for integrated exposure and biomarker analyses in future developmental toxicology research.

### 4.3 Strengths and limitations

Our study has several strengths and novel features. First, we are among the first to study maternal PFAS exposure interactions with estradiol and progesterone in relation to developmental delay, using a large prospective cohort (*n* = 543) with repeated ASQ-3 measurements (3–60 months) to strengthen temporal inference and reduce recall bias [29,58]. Moreover, we identified hormone strata via LPA by combining estradiol and progesterone, capturing effect modification more comprehensively than single hormones alone [26]. Third, we employed the GWQS approach to account for the practical context of concurrent exposure to various PFAS substances and to measure their combined impacts, an area that has received insufficient investigation in child cohorts [85,86]. Finally, exploratory molecular docking of PFAS to ERα/β and PR provided preliminary mechanistic plausibility for observed associations.

Nonetheless, this study has certain limitations. First, while the high-estradiol and progesterone group was relatively small (17.0%, *n* = 92), our longitudinal repeated measures design and GLMM analysis enhanced statistical power to detect significant associations within this stratum. However, this sample size restricted our ability to perform further stratification (e.g., child sex) without compromising statistical robustness. Second, residual confounding due to unmeasured factors, including maternal psychological stress [87], paternal PFAS exposure [7], and specific dietary patterns [15], may bias the observed associations. Moreover, molecular docking supports mechanistic plausibility but should be considered preliminary, as *in silico* predictions cannot fully capture *in vivo* toxicokinetics or the dynamic and competitive nature of receptor binding [24,79]. Finally, hormones were measured only once at delivery, a single timepoint that cannot fully reflect their dynamic fluctuations throughout gestation. However, this sampling window coincides with critical late-pregnancy neurodevelopmental events (e.g., synaptogenesis and the onset of myelination) [88], and thus still yields biologically meaningful data on fetal endocrine exposure during this key period. Future research should consider larger, multi-center cohorts with repeated exposure and biomarker measurements, as well as experimental validation, to better establish causal pathways and inform early-life environmental health interventions.

## 5 Conclusion

In this prospective birth cohort, prenatal PFAS exposure was linked to early neurodevelopmental delays, with associations differing by subclass and further modified by cord estradiol and progesterone profiles. Larger prospective studies with longer follow-up, repeated prenatal hormone measurements, and mechanistic experiments are needed to validate these findings and clarify endocrine-related pathways.

## CRediT authorship contribution statement

**Haochen Lin:** Writing – review & editing, Writing – original draft, Visualization, Formal analysis, Data curation, Conceptualization. **Qiong Zhang:** Writing – original draft, Methodology, Formal analysis, Data curation. **Xiaona Chen:** Methodology, Data curation, Conceptualization. **Yang Zhou:** Formal analysis, Data curation, Conceptualization. **Guangzhen Liu:** Data curation, Conceptualization. **Yanying Wu:** Data curation, Conceptualization. **Feifei Qu:** Visualization, Formal analysis. **Longshen Fan:** Visualization, Formal analysis. **Dan Cai:** Methodology, Conceptualization. **Guanghui Dong:** Funding acquisition, Conceptualization. **Shaoya Huang:** Supervision, Resources, Data curation. **Xiaowen Zeng:** Writing – review & editing, Supervision, Resources, Project administration, Funding acquisition, Data curation, Conceptualization.

## Declaration of competing interest

The authors declare that they have no competing interests.
