# Supplementary material for: Prenatal exposure to perfluoroalkyl sulfonic and carboxylic acids and neurodevelopmental delay in children up to 5 years old: Effect modification by progesterone and estradiol
Source: Eco Environ Health. 2026 Mar 24;5(2):100238. doi: 10.1016/j.eehl.2026.100238 (PMC13090710; doi:10.1016/j.eehl.2026.100238)
Supplement: Multimedia component 1 [file mmc1.docx]

***Supplementary Materials***

# Prenatal exposure to perfluoroalkyl sulfonic and carboxylic acids and neurodevelopmental delay in children up to 5 years old: Effect modification by progesterone and estradiol

Haochen Lin ^a, 1^, Qiong Zhang ^a, 1^, Xiaona Chen ^b, 1^, Yang Zhou ^a^, Guangzhen Liu ^b^, Yanying Wu ^b^, Feifei Qu ^a^, Longshen Fan ^a^, Dan Cai ^c^, Guanghui Dong ^a,^ *, Shaoya Huang ^b,^ *, Xiaowen Zeng ^a,^ *

^a^ *Joint International Research Laboratory of Environment and Health, Ministry of Education, Guangdong Provincial Engineering Technology Research Center of Environmental Pollution and Health Risk Assessment, Department of Occupational and Environmental Health, School of Public Health, Sun Yat-sen University, Guangzhou 510080, China*

^b^ *Maoming Maternal and Child Health Hospital, Maoming 525000, China*

^c^ *Department of Public Health and Preventive Medicine, School of Medicine, Jinan University, Guangzhou 510632, China*

* Corresponding author.

*E-mail addresses:* donggh5@mail.sysu.edu.cn (G. Dong), hshaoya@163.com (S. Huang), zxw63@mail.sysu.edu.cn (X. Zeng).

^1^ These authors contributed equally to this work.

**Content:**

**Table S1-S18**

**Figure S1-S7**

**Table S1.** Demographic characteristics of the study and excluded population.

| **variable** | **study population (*n* = 543)** | **excluded population (*n* = 487)** | ***P* - value** |
| --- | --- | --- | --- |
| **Maternal age (years)** | 28.80 ± 5.53 | 28.82 ±5.01 | 0.966 |
| Missing | 6 (1.10%) | 1 (0.21%) |  |
| **Pre-BMI (kg/m^2^)** | 21.16 ± 4.79 | 21.20 ± 4.84 | 0.901 |
| Missing | 59 (10.87%) | 11 (2.26%) |  |
| **Delivery method** |  |  | 0.128 |
| vaginal | 308 (56.72%) | 289 (59.34%) |  |
| cesarean | 231 (42.54%) | 198 (40.66%) |  |
| Missing | 5 (0.74%) | - |  |
| **Parity** |  |  | 0.081 |
| Primipara | 307 (56.54%) | 246 (50.51%) |  |
| Multipara | 232 (42.73%) | 233 (47.84%) |  |
| Missing | 4 (0.73%) | 8 (1.64%) |  |
| **Gestational age (weeks)** | 37.00 ± 2.90 | 37.08 ± 3.11 | 0.672 |
| Missing | 6 (1.10%) | 6 (1.23%) |  |
| **Maternal education** |  |  | 0.291 |
| ≤high school | 285 (52.49%) | 272 (55.85%) |  |
| >high school | 212 (39.04%) | 185 (37.99%) |  |
| Missing | 46 (8.47%) | 30 (6.16%) |  |
| **Family income (CNY/year)** |  |  | 0.924 |
| <30,000 | 143 (26.34%) | 126 (25.87%) |  |
| 30,000−100,000 | 209 (38.49%) | 181 (37.17%) |  |
| >100,000 | 88 (16.21%) | 86 (17.66%) |  |
| Missing | 103 (18.97%） | 94 (19.30%) |  |
| **Nutrient supplementation during pregnancy** |  |  | 0.093 |
| Yes | 74 (13.63%) | 87 (17.86%) |  |
| No | 412 (78.87%) | 361 (74.13%) |  |
| Missing | 57 (7.50%） | 39 (8.01%) |  |
| **Infant sex** |  |  | 0.136 |
| male | 294 (54.14%) | 275 (56.47%) |  |
| female | 245 (45.12%) | 212 (43.53%) |  |
| Missing | 4 (0.74%) | - |  |
| **Birth weight (g)** | 2767 ± 630 | 2817 ± 619 | 0.201 |
| Missing | 4 (0.74%) | - |  |
| **Breastfeeding duration** |  |  | < 0.001 |
| 0 month | 284 (52.30%) | 298 (61.19%) |  |
| ≥6 months | 123 (22.65%) | 65 (13.35%) |  |
| <6 months | 76 (14.00%) | 57 (11.70%) |  |
| Missing | 60 (11.05%) | 67 (13.76%) |  |

For continuous variables, use mean (SD); for categorical variables, use *n*(%). Pre-BMI, body mass index before pregnancy; CNY, Chinese Yuan. *P* for difference between the study population (*n* = 543) and the excluded population (*n* = 487) according to the χ2 test for nominal variables and Wilcoxon rank sum test for continuous variables.

Table S2. Abbreviation, full name, detection rate, limit of detection (LOD), and maternal serum blood concentrations of 32 kinds of PFAS in our study (*n* = 543).

| Abbreviation | Full name | Detection rate (%) | LOD (ng/mL) |
| --- | --- | --- | --- |
| HFPO-DA | Hexafluoro-1-propylene oxide dimer acid | 0.18% | 0.5025 |
| PFBS | Perfluoro-1-butansulfonic acid | 4.79% | 0.0095 |
| **PFBA** | Perfluoro-n-butanoic acid | 78.08% | 0.0081 |
| PFPeS | Perfluoro-1-pentanesulfonic acid | 7.92% | 0.0029 |
| PFPeA | Perfluoro-n-pentanoic acid | 39.96% | 0.0179 |
| **PFHxA** | Perfluoro-n-hexanoic acid | 79.37% | 0.0035 |
| **linear-PFHxS** | linear perfluoro-1-hexane sulfonate | 98.90% | 0.0011 |
| **br-PFHxS** | Sum of all branched isomers PFHxS | 53.22% | 0.0011 |
| **PFHpS** | Perfluoro-1-heptanesulfonic acid | 85.45% | 0.0021 |
| PFHpA | Perfluoro-n-heptanoic acid | 56.17% | 0.0016 |
| **linear PFOS** | Perfluoro-1-octane sulfonate | 99.82% | 0.0016 |
| **br-PFOS** | branched isomers PFOS | 100% | 0.0003 |
| **PFOA** | Perfluoro-n-octanoic acid | 99.45% | 0.0025 |
| FOSA | Perfluoro-1-octanesulfonamide | 34.99% | 0.0011 |
| N-MeFOSAA | N-methylperfluoro-1-octanesulfonamidoacetic acid | 0.18% | 0.0044 |
| N-EtFOSAA | N-ethylperfluoro-1-octanesulfonamidoacetic acid | 0.37% | 0.0016 |
| **6:2 Cl-PFESA** | 6:2 chlorinated polyfluorinated ether sulfonate acid | 99.82% | 0.0018 |
| **8:2 Cl-PFESA** | 8:2 chlorinated polyfluorinated ether sulfonate acid | 78.08% | 0.0015 |
| PFNS | Perfluoro-1-nonane sulfonic acid | 2.76% | 0.006 |
| **PFNA** | Perfluorononanoic acid | 99.82% | 0.0024 |
| PFDS | Perfluoro-1-decane sulfonic acid | 4.05% | 0.0038 |
| **PFDA** | Perfluoro-n-decanoic acid | 99.82% | 0.0048 |
| **PFUnDA** | Perfluoro-n-undecanoic acid | 99.26% | 0.0049 |
| **PFDoDA** | Perfluoro-n-dodecanoic acid | 77.16% | 0.0057 |
| **PFTrDA** | Perfluoro-n-tridecanoic acid | 99.26% | 0.0106 |
| PFTeDA | Perfluoro-n-tetradecanoic acid | 63.90% | 0.0036 |
| 4:2FTSA | 1H, 1H, 2H, 2H-perfluoro-1-hexanesulfonic acid | 0 | 0.0073 |
| 6:2FTSA | 1H, 1H, 2H, 2H-perfluoro-1-octanesulfonic acid | 6.81% | 0.0106 |
| 8:2FTSA | 1H, 1H, 2H, 2H-perfluoro-1-decanesulfonic acid | 0.18% | 0.0017 |

The PFAS compounds marked in bold represent the target compounds analyzed in this study.

| **Table S3.** Repeated measures of neuropsychologicaldevelopments at different developmental stages (*n* = 543, 1983 repeated measures). | | | | | | | | | |
| --- | --- | --- | --- | --- | --- | --- | --- | --- | --- |
| Developmental subscales | ASQ-3 scores (Mean ± SD) | | | | | | |  |  |
|  | 3 months  (*n* = 416） | 6 months  (*n* = 385） | 12 months  (*n* = 294） | 18 months  (*n* = 259） | 24 months  (*n* = 238） | 36 months  (*n* = 157） | 48 months  (*n* = 92） | 60 months  (*n* = 142) | |
| Communication | 44.05 ± 13.14 | 45.25 ± 9.84 | 44.82 ± 11.99 | 37.65 ± 12.30 | 45.61 ± 15.26 | 50.57 ± 11.87 | 53.04 ± 9.02 | 52.18 ±7.91 | |
| Gross motor function | 46.57 ± 10.71 | 38.20 ± 12.49 | 46.00 ± 14.50 | 54.44 ± 7.92 | 55.47 ± 7.86 | 54.25 ± 8.85 | 56.63 ± 7.92 | 58.56 ± 4.02 | |
| Fine motor function | 37.57 ± 11.48 | 50.57 ± 10.47 | 47.01 ± 9.08 | 49.21 ± 9.71 | 48.63 ± 7.30 | 45.35 ± 14.26 | 48.32 ± 11.35 | 52.64 ± 8.58 | |
| Problem-solving ability | 40.90 ± 9.85 | 49.00 ± 10.36 | 44.24 ± 12.05 | 47.81 ± 8.69 | 51.53 ± 9.41 | 45.03 ± 12.68 | 53.26 ± 9.76 | 54.96 ± 7.16 | |
| Personal-social skills | 42.17 ± 10.39 | 40.86 ± 11.79 | 38.95 ± 10.83 | 48.50 ± 8.74 | 43.47 ± 9.87 | 45.46 ± 10.50 | 53.70 ± 6.46 | 58.35 ± 3.89 | |

ASQ-3: the Ages and Stages Questionnaires, 3rd edition.

Table S4. Crude ORs (95% CIs) per unit increase in prenatal PFAS levels for developmental delay in children aged 3–60 months: A longitudinal analysis (*n* = 543).

| Serum PFAS (ng/mL) | ASQ | Communication | Gross motor function | Fine motor  function | Problem-solving ability | Personal-social skills |
| --- | --- | --- | --- | --- | --- | --- |
|  | Crude ORs (95% CIs) | | | | | |
| ***PFCAs ^a^*** |  | | | | | |
| PFBA | 1.039 (0.965, 1.119) | 1.160 (0.941, 1.430) | 1.033 (0.898, 1.189) | **0.990 (0.986, 0.994)** | 0.961 (0.745, 1.239) | 1.010 (0.798, 1.278) |
| PFHxA | 0.983 (0.878, 1.101) | 1.125 (0.828, 1.529) | 0.969 (0.782, 1.201) | 0.866 (0.630, 1.191) | 0.846 (0.564, 1.269) | 1.011 (0.705, 1.450) |
| PFOA | **1.264 (1.012, 1.580)** | 1.279 (0.719, 2.276) | 1.169 (0.788, 1.735) | 1.144 (0.602, 2.172) | 1.237 (0.556, 2.749) | 1.459 (0.722, 2.949) |
| PFNA | 1.236 (0.905, 1.688) | 1.273 (0.537, 3.021) | 1.019 (0.571, 1.817) | 1.312 (0.245, 7.022) | 1.071 (0.360, 3.188) | 1.272 (0.456, 3.547) |
| PFDA | **1.312 (1.002, 1.716)** | 1.219 (0.587, 2.530) | 1.037 (0.628, 1.713) | 1.652 (0.952, 2.867) | 1.212 (0.457, 3.213) | 1.290 (0.540, 3.079) |
| PFUnDA | **1.287 (1.000, 1.657)** | 1.343 (0.669, 2.695) | 1.091 (0.693, 1.719) | 1.344 (0.634, 2.847) | 1.138 (0.472, 2.748) | 1.217 (0.543, 2.728) |
| PFDoDA | 0.891 (0.778, 1.021) | 0.908 (0.629, 1.312) | 0.806 (0.626, 1.036) | **0.873 (0.870, 0.876)** | 0.890 (0.552, 1.436) | 0.895 (0.584, 1.373) |
| PFTrDA | 0.986 (0.796, 1.222) | 0.962 (0.542, 1.708) | 0.871 (0.588, 1.292) | 1.017 (0.546, 1.894) | 0.982 (0.453, 2.129) | 1.015 (0.510, 2.023) |
| GWQS index1 | **1.351 (1.100, 1.659)** | **1.704 (1.056, 2.749)** | 1.374 (0.893, 2.113) | 1.195 (0.658, 2.169) | 1.023 (0.458, 2.286) | **1.491 (1.482, 1.499)** |
| ***PFSAs ^b^*** |  |  |  |  |  |  |
| linear-PFHxS | 1.173 (0.976, 1.409) | 1.177 (0.715, 1.938) | 1.018 (0.726, 1.425) | 1.086 (0.648, 1.821) | 1.023 (0.548, 1.907) | 1.209 (0.657, 2.227) |
| br-PFHxS | 1.048 (0.916, 1.199) | 0.996 (0.687, 1.444) | 0.897 (0.687, 1.170) | 0.890 (0.670, 1.184) | 1.097 (0.683, 1.761) | 1.113 (0.729, 1.700) |
| 6:2 Cl-PFESA | 1.201 (0.959, 1.504) | 1.418 (0.758, 2.651) | 1.259 (0.824, 1.924) | 1.191 (0.748, 1.896) | 1.146 (0.509, 2.582) | 1.080 (0.531, 2.194) |
| linear-PFOS | 1.175 (0.962, 1.435) | 1.510 (0.860, 2.651) | 1.152 (0.791, 1.677) | 1.194 (0.792, 1.801) | 1.137 (0.555, 2.330) | **1.233 (1.228, 1.238)** |
| br-PFOS | 1.131 (0.917, 1.395) | 1.381 (0.770, 2.476) | 1.083 (0.729, 1.610) | 1.004 (0.553, 1.825) | 1.095 (0.516, 2.323) | 1.185 (0.602, 2.333) |
| PFHpS | 0.929 (0.835, 1.034) | 0.958 (0.715, 1.284) | 0.936 (0.765, 1.146) | **0.889 (0.885, 0.893)** | 0.890 (0.612, 1.295) | **0.871 (0.868, 0.874)** |
| 8:2 Cl-PFESA | 1.049 (0.908, 1.212) | 1.131 (0.765, 1.674) | 1.039 (0.793, 1.362) | **1.033 (1.028, 1.038)** | 1.119 (0.661, 1.893) | 0.948 (0.601, 1.497) |
| GWQS index2 | 1.072 (0.849, 1.353) | 1.260 (0.780, 2.035) | 1.117 (0.811, 1.538) | 1.115 (0.804, 1.547) | 0.993 (0.392, 2.513) | 1.124 (0.521, 2.425) |

*^a^* Sum of PFBA, PFHxA, PFOA, PFNA, PFDA, PFUnDA, PFDoDA, and PFTrDA.

*^b^* Sum of linear-PFHxS, br-PFHxS, 6:2 Cl-PFESA, linear-PFOS, br-PFOS, PFHpS, and 8:2 Cl-PFESA.

GWQS index 1 and GWQS index 2 were respectively created using all types of PFCAs and PFSAs to reflect their mixture exposure levels.

Odds ratios and 95% confidence intervals are reported to three decimal places due to numerically close confidence interval bounds. Values in bold represent *P* <0.05.

Table S5. Weights of individual PFAS in PFCAs and PFSAs groups in grouped weighted quantile sum (GWQS) regression models (*n* = 543).

| PFAS | ASQ | Communication | Gross motor function | Fine motor function | Problem-solving ability | Personal-social skills |
| --- | --- | --- | --- | --- | --- | --- |
| Σ PFCAs *^a^* | 1.00 | 1.00 | 1.00 | 1.00 | 1.00 | 1.00 |
| PFBA | 0.44 | 0.68 | 0.34 | 0.16 | 0.20 | 0.34 |
| PFHxA | 0.05 | 0.08 | 0.14 | 0.06 | 0.02 | 0.27 |
| PFOA | 0.36 | 0.11 | 0.24 | 0.17 | 0.28 | 0.33 |
| PFNA | 0.00 | 0.00 | 0.00 | 0.03 | 0.02 | 0.02 |
| PFDA | 0.03 | 0.03 | 0.07 | 0.36 | 0.26 | 0.02 |
| PFUnDA | 0.11 | 0.08 | 0.18 | 0.13 | 0.01 | 0.01 |
| PFDoDA | 0.00 | 0.00 | 0.01 | 0.01 | 0.00 | 0.00 |
| PFTrDA | 0.00 | 0.02 | 0.02 | 0.07 | 0.20 | 0.00 |
| Σ PFSAs *^b^* | 1.00 | 1.00 | 1.00 | 1.00 | 1.00 | 1.00 |
| linear-PFHxS | 0.05 | 0.01 | 0.02 | 0.05 | 0.00 | 0.30 |
| br-PFHxS | 0.27 | 0.03 | 0.02 | 0.04 | 0.35 | 0.57 |
| 6:2 Cl-PFESA | 0.14 | 0.15 | 0.85 | 0.03 | 0.08 | 0.00 |
| linear-PFOS | 0.50 | 0.53 | 0.00 | 0.86 | 0.31 | 0.03 |
| br-PFOS | 0.01 | 0.15 | 0.09 | 0.00 | 0.07 | 0.05 |
| PFHpS | 0.00 | 0.01 | 0.00 | 0.01 | 0.08 | 0.00 |
| 8:2 Cl-PFESA | 0.03 | 0.12 | 0.00 | 0.01 | 0.11 | 0.06 |

*^a^* Sum of PFBA, PFHxA, PFOA, PFNA, PFDA, PFUnDA, PFDoDA, and PFTrDA.

*^b^* Sum of linear-PFHxS, br-PFHxS, 6:2 Cl-PFESA, linear-PFOS, br-PFOS, PFHpS, and 8:2 Cl-PFESA.

The models were adjusted for pre-pregnancy BMI, parity, maternal education, family income, nutrient supplementation during pregnancy, infant sex, and maternal age.

Table S6. Fit Statistics and Classification Coefficients.

| K | AIC | BIC | Entropy | LMR*P* | Category Probabilities |
| --- | --- | --- | --- | --- | --- |
| 1 | 11261.02 | 11283.389 | - | - | - |
| **2** | **2278.83** | **2307.24** | **0.89** | **<0.001** | **0.82/0.18** |
| 3 | 2242.16 | 2282.75 | 0.81 | 0.1929 | 0.70/0.14/0.15 |
| 4 | 2189.40 | 2242.17 | 0.86 | 0.0005 | 0.178/0.055/0.667/0.099 |
| 5 | 2169.41 | 2234.35 | 0.86 | 0.0139 | 0.153/0.029/0.124/0.061/0.632 |

K = number of classes; AIC = Akaike information criterion; BIC = Bayesian information criterion; LMR-LRT = Lo-Mendell-Rubin test; Bold font indicates selected model.

Table S7. Generalized linear mixed model for the association between developmental delay and maternal serum PFAS (ln ng/ml) based on the percentile distribution of umbilical cord progesterone and estradiol (n = 543).

| Ln-PFAS | Hormone levels | ASQ | Communication | Gross motor function | Fine motor function | Problem-solving ability | Personal-social skills |
| --- | --- | --- | --- | --- | --- | --- | --- |
|  |  | OR (95% CI) | OR (95% CI) | OR (95% CI) | OR (95% CI) | OR (95% CI) | OR (95% CI) |
| ***PFCAs*** |  |  |  |  |  |  |  |
| PFBA | low | 1.008 (0.933, 1.090) | 1.054 (0.905, 1.228) | 1.055 (0.913, 1.218) | 0.965 (0.844, 1.103) | 0.93 (0.519, 1.667) | 1.060 (0.829, 1.355) |
|  | high | 1.157 (0.931, 1.438) | 1.869 (0.978, 3.571) | 0.852 (0.607, 1.195) | 1.079 (0.668, 1.744) | 0.938 (0.205, 4.296) | 0.865 (0.474, 1.581) |
| PFHxA | low | 0.969 (0.858, 1.095) | 1.125 (0.895, 1.413) | 0.903 (0.719, 1.133) | 0.848 (0.685, 1.050) | 0.61 (0.407, 0.915) | 1.041 (0.812, 1.335) |
|  | high | 1.086 (0.813, 1.452) | 0.937 (0.59, 1.488) | 1.030 (0.604, 1.755) | 0.848 (0.490, 1.468) | 0.713 (0.289, 1.758) | 0.526 (0.275, 1.007) |
| PFOA | low | 1.230 (0.958, 1.580) | 1.031 (0.655, 1.621) | 1.030 (0.682, 1.554) | 1.156 (0.755, 1.770) | 0.959 (0.495, 1.857) | 1.591 (0.958, 2.643) |
|  | high | 1.292 (0.797, 2.094) | 2.276 (0.937, 5.526) | 1.309 (0.559, 3.064) | 0.992 (0.416, 2.363) | 0.736 (0.238, 2.278) | 0.721 (0.324, 1.606) |
| PFNA | low | 1.187 (0.836, 1.684) | 1.285 (0.622, 2.657) | 1.088 (0.569, 2.080) | 1.654 (0.836, 3.271) | 1.260 (0.568, 2.797) | 1.130 (0.559, 2.286) |
|  | high | 1.256 (0.512, 3.081) | 0.744 (0.162, 3.412) | 0.709 (0.131, 3.834) | 0.474 (0.070, 3.233) | 0.526 (0.067, 4.114) | 0.612 (0.099, 3.783) |
| PFDA | low | 1.292 (0.960, 1.739) | 1.083 (0.613, 1.914) | 1.047 (0.623, 1.76) | **2.035 (1.188, 3.486)** | 1.506 (0.695, 3.263) | 1.192 (0.571, 2.487) |
|  | high | 1.171 (0.551, 2.485) | 0.843 (0.255, 2.793) | 0.540 (0.136, 2.153) | **0.257 (0.053, 1.236)** | 0.123 (0.005, 2.854) | 0.451 (0.068, 2.999) |
| PFUnDA | low | 1.228 (0.918, 1.642) | 1.283 (0.702, 2.348) | 1.039 (0.625, 1.729) | **1.679 (0.965, 2.921)** | 1.040 (0.390, 2.768) | 0.980 (0.453, 2.119) |
|  | high | 1.297 (0.696, 2.418) | 1.340 (0.445, 4.032) | 1.426 (0.414, 4.906) | **0.565 (0.246, 1.300)** | 1.963 (0.128, 30.208) | 0.491 (0.083, 2.893) |
| PFDoDA | low | 0.900 (0.778, 1.041) | 0.923 (0.690, 1.236) | 0.784 (0.597, 1.030) | **0.922 (0.722, 1.178)** | 0.797 (0.532, 1.193) | 0.905 (0.668, 1.225) |
|  | high | 0.884 (0.631, 1.239) | 0.831 (0.464, 1.489) | 0.879 (0.471, 1.642) | **0.421 (0.213, 0.833)** | 0.256 (0.057, 1.161) | 0.435 (0.191, 0.991) |
| PFTrDA | low | 0.943 (0.748, 1.189) | 0.761 (0.509, 1.138) | 0.780 (0.519, 1.171) | **1.041 (1.039, 1.043)** | 0.840 (0.833, 0.846) | 1.029 (0.492, 2.150) |
|  | high | 0.892 (0.467, 1.702) | 1.037 (0.395, 2.727) | 0.849 (0.245, 2.945) | **0.402 (0.401, 0.403)** | 0.240 (0.032, 1.795) | 0.661 (0.087, 5.025) |
| ***PFSAs*** |  |  |  |  |  |  |  |
| PFHpS | low | **0.881 (0.787, 0.987)** | 0.847 (0.691, 1.039) | **0.786 (0.653, 0.947)** | 0.901 (0.736, 1.104) | 0.846 (0.679, 1.056) | 0.835 (0.674, 1.035) |
|  | high | **1.305 (0.942, 1.807)** | 1.268 (0.778, 2.066) | **2.628 (1.184, 5.833)** | 1.337 (0.687, 2.604) | 1.175 (0.608, 2.270) | 1.581 (0.766, 3.260) |
| linear-PFHxS | low | 1.124 (0.929, 1.359) | 1.179 (0.813, 1.711) | 0.925 (0.663, 1.290) | 1.259 (0.857, 1.850) | **0.567 (0.561, 0.573)** | 1.136 (0.746, 1.730) |
|  | high | 1.378 (0.799, 2.379) | 1.164 (0.482, 2.809) | 1.844 (0.635, 5.352) | 0.781 (0.289, 2.113) | **0.914 (0.908, 0.921)** | 0.855 (0.283, 2.585) |
| br-PFHxS | low | 1.086 (0.946, 1.247) | 1.051 (0.816, 1.354) | 0.834 (0.643, 1.083) | 0.827 (0.650, 1.052) | 1.070 (0.811, 1.412) | 0.960 (0.636, 1.451) |
|  | high | 0.778 (0.490, 1.235) | 0.522 (0.235, 1.159) | 0.562 (0.223, 1.414) | 0.352 (0.102, 1.215) | 0.344 (0.1, 1.187) | 0.430 (0.083, 2.233) |
| 6:2Cl-PFESA | low | 1.152 (0.892, 1.489) | 1.28 (0.772, 2.124) | 1.128 (0.705, 1.805) | 1.378 (0.856, 2.217) | 0.914 (0.908, 0.919) | 1.005 (0.509, 1.983) |
|  | high | 1.260 (0.741, 2.143) | 1.556 (0.635, 3.813) | 1.903 (0.745, 4.859) | 1.283 (0.479, 3.439) | 0.785 (0.137, 4.499) | 0.459 (0.069, 3.042) |
| linear-PFOS | low | 1.072 (0.863, 1.333) | 1.179 (0.761, 1.826) | **0.903 (0.656, 1.243)** | 1.161 (0.786, 1.716) | **0.920 (0.641, 1.319)** | 0.936 (0.621, 1.410) |
|  | high | 1.590 (0.930, 2.719) | 2.839 (1.126, 7.161) | **4.758 (1.962, 11.535)** | 2.505 (0.913, 6.874) | **3.472 (1.377, 8.753)** | 2.397 (0.895, 6.419) |
| br-PFOS | low | 1.022 (0.812, 1.286) | **1.071 (0.705, 1.626)** | **0.801 (0.556, 1.153)** | 0.900 (0.604, 1.340) | 0.754 (0.374, 1.522) | 0.875 (0.553, 1.386) |
|  | high | 1.682 (0.970, 2.917) | **2.857 (1.182, 6.910)** | **4.148 (1.479, 11.627)** | 1.678 (0.629, 4.473) | 0.413 (0.024, 7.029) | 2.356 (0.753, 7.376) |
| 8:2Cl-PFESA | low | 0.991 (0.846, 1.160) | 1.034 (0.772, 1.384) | 0.899 (0.675, 1.197) | 0.983 (0.767, 1.260) | 1.775 (0.947, 3.328) | 0.988 (0.717, 1.360) |
|  | high | 1.173 (0.842, 1.634) | 1.284 (0.755, 2.181) | 1.064 (0.603, 1.880) | 1.059 (0.606, 1.851) | 1.051 (0.458, 2.413) | 0.796 (0.404, 1.569) |

Values in bold represent statistically significant interactions (*P* < 0.05) between PFAS exposure and progesterone and estradiol level strata. Odds ratios and 95% confidence intervals are reported to three decimal places due to numerically close confidence interval bounds. The models were adjusted for pre-pregnancy BMI, parity, maternal education, family income, nutrient supplementation during pregnancy, infant sex, and maternal age.

Table S8. Adjusted ORs (95% CIs) per unit increase in individual PFAS levels and GWQS index for developmental delay in children aged 3–60 months: A longitudinal analysis (additionally adjusting for delivery method, birthweight, and breastfeeding duration).

| Serum PFAS (ng/mL) | ASQ | Communication | Gross motor function | Fine motor  function | Problem-solving ability | Personal-social skills |
| --- | --- | --- | --- | --- | --- | --- |
|  | Adjusted ORs (95% CIs) | | | | | |
| ***PFCAs ^a^*** |  |  |  |  |  |  |
| PFBA | 1.018 (0.950, 1.091) | 1.120 (0.976, 1.285) | 0.996 (0.887, 1.118) | 0.996 (0.891, 1.113) | 0.923 (0.734, 1.161) | 0.961 (0.837, 1.103) |
| PFHxA | 0.991 (0.891, 1.101) | 1.125 (0.925, 1.370) | 1.023 (0.859, 1.218) | 0.946 (0.779, 1.149) | **0.761 (0.590, 0.981)** | **0.998 (0.995, 1.000)** |
| PFOA | 1.146 (0.928, 1.414) | 1.118 (0.780, 1.600) | 0.961 (0.708, 1.303) | 1.162 (0.813, 1.659) | 0.890 (0.290, 2.737) | 1.323 (0.865, 2.024) |
| PFNA | 1.074 (0.780, 1.478) | 1.017 (0.559, 1.850) | 0.831 (0.522, 1.323) | 1.352 (0.772, 2.367) | 0.459 (0.160, 1.314) | 0.891 (0.511, 1.556) |
| PFDA | 1.195 (0.913, 1.564) | 1.060 (0.628, 1.791) | 0.933 (0.604, 1.440) | 1.452 (0.911, 2.314) | 0.777 (0.443, 1.365) | 0.916 (0.552, 1.519) |
| PFUnDA | 1.112 (0.857, 1.443) | 1.075 (0.662, 1.744) | 0.900 (0.599, 1.352) | 1.295 (0.793, 2.115) | 1.092 (0.607, 1.964) | 0.897 (0.560, 1.438) |
| PFDoDA | 0.935 (0.819, 1.068) | 0.970 (0.760, 1.238) | 0.894 (0.727, 1.100) | 0.890 (0.708, 1.120) | 0.802 (0.606, 1.059) | 1.007 (0.786, 1.290) |
| PFTrDA | 0.931 (0.755, 1.149) | 0.860 (0.580, 1.275) | 0.883 (0.646, 1.208) | 1.073 (0.732, 1.575) | 0.984 (0.655, 1.478) | 0.843 (0.573, 1.240) |
| GWQS index1 | 1.111 (0.884, 1.396) | 1.324 (0.930, 1.885) | 1.027 (0.723, 1.457) | 1.106 (0.778, 1.572) | 0.809 (0.546, 1.199) | 1.056 (0.723, 1.544) |
| ***PFSAs ^b^*** |  |  |  |  |  |  |
| linear-PFHxS | **1.202 (1.011, 1.430)** | 1.235 (0.894, 1.705) | 0.986 (0.747, 1.301) | 1.199 (0.878, 1.638) | 0.943 (0.637, 1.395) | 1.103 (0.793, 1.534) |
| br-PFHxS | 1.084 (0.955, 1.232) | 1.036 (0.816, 1.314) | 0.877 (0.698, 1.102) | 0.870 (0.684, 1.107) | 1.034 (0.789, 1.356) | 1.079 (0.855, 1.361) |
| 6:2 Cl-PFESA | 1.092 (0.872, 1.367) | 1.217 (0.792, 1.872) | 1.059 (0.732, 1.533) | 1.272 (0.855, 1.893) | 1.200 (0.291, 4.946) | 0.911 (0.567, 1.463) |
| linear-PFOS | 1.072 (0.879, 1.309) | 1.289 (0.874, 1.902) | 1.036 (0.748, 1.436) | 1.246 (0.888, 1.749) | 1.135 (0.754, 1.710) | 1.245 (0.835, 1.855) |
| br-PFOS | 1.062 (0.862, 1.308) | 1.294 (0.862, 1.943) | 0.961 (0.680, 1.358) | 1.140 (0.757, 1.716) | 1.004 (0.995, 1.013) | 1.073 (0.689, 1.668) |
| PFHpS | 0.964 (0.870, 1.068) | 1.019 (0.834, 1.244) | 0.966 (0.814, 1.146) | 0.988 (0.828, 1.179) | 0.802 (0.583, 1.103) | 0.854 (0.694, 1.052) |
| 8:2 Cl-PFESA | 1.014 (0.885, 1.162) | 1.109 (0.855, 1.438) | 0.943 (0.763, 1.165) | 0.990 (0.774, 1.267) | 1.000 (0.766, 1.306) | 0.937 (0.728, 1.206) |
| GWQS index2 | 1.017 (0.831,1.244) | 1.111 (0.799, 1.544) | 0.990 (0.716, 1.369) | 1.154 (0.834, 1.596) | 0.999 (0.683, 1.461) | 1.012 (0.708, 1.446) |

*^a^* Sum of PFBA, PFHxA, PFOA, PFNA, PFDA, PFUnDA, PFDoDA, and PFTrDA.

*^b^* Sum of linear-PFHxS, br-PFHxS, 6:2 Cl-PFESA, linear-PFOS, br-PFOS, PFHpS, and 8:2 Cl-PFESA.

GWQS index 1 and GWQS index 2 were respectively created using all types of PFCAs and PFSAs to reflect their mixture exposure levels.

Odds ratios and 95% confidence intervals are reported to three decimal places due to numerically close confidence interval bounds. Values in bold represent *P* < 0.05. The models were adjusted for pre-BMI, parity, maternal education, family income, nutrient supplementation during pregnancy, infant sex, maternal age, delivery method, birthweight, and breastfeeding duration.

Table S9. Adjusted ORs (95% CIs) per unit increase in individual PFAS levels and WQS index for developmental delay in children aged 3–60 months: A longitudinal analysis (using complete data).

| Serum PFAS (ng/mL) | ASQ | Communication | Gross-motor function | Fine motor  function | Problem-solving ability | Personal-social skills |
| --- | --- | --- | --- | --- | --- | --- |
|  | Adjusted ORs (95% CIs) | | | | | |
| ***PFCAs ^a^*** |  |  |  |  |  |  |
| PFBA | 1.006 (0.915, 1.106) | 1.148 (0.948, 1.390) | 1.009 (0.861, 1.183) | 0.919 (0.789, 1.070) | 0.870 (0.703, 1.077) | 0.967 (0.820, 1.141) |
| PFHxA | 1.006 (0.869, 1.164) | 1.106 (0.854, 1.431) | 0.906 (0.717, 1.144) | 0.853 (0.651, 1.116) | **0.743 (0.558, 0.989)** | 1.002 (0.744, 1.349) |
| PFOA | **1.356 (1.020, 1.803)** | 1.364 (0.737, 2.523) | 1.063 (0.703, 1.607) | 1.262 (0.739, 2.155) | 1.153 (0.655, 2.030) | 1.415 (0.910, 2.199) |
| PFNA | 1.211 (0.807, 1.815) | 1.180 (0.460, 3.026) | 0.864 (0.464, 1.607) | 1.948 (0.830, 4.570) | **1.168 (1.165, 1.171)** | 1.237 (0.640, 2.390) |
| PFDA | 1.357 (0.957, 1.925) | 1.159 (0.510, 2.633) | 1.029 (0.591, 1.791) | **1.927 (1.074, 3.457)** | 1.026 (0.566, 1.857) | 1.159 (0.655, 2.051) |
| PFUnDA | 1.408 (0.989, 2.003) | 1.567 (0.602, 4.077) | 1.251 (0.711, 2.200) | 1.841 (0.958, 3.541) | 0.924 (0.528, 1.619) | 1.270 (0.679, 2.375) |
| PFDoDA | 0.959 (0.809, 1.137) | 1.010 (0.649, 1.572) | 0.829 (0.625, 1.100) | 1.018 (0.777, 1.333) | **0.908 (0.905, 0.910)** | 1.012 (0.741, 1.382) |
| PFTrDA | 1.048 (0.788, 1.395) | 1.030 (0.588, 1.803) | 0.855 (0.550, 1.329) | 1.291 (0.774, 2.154) | 0.830 (0.515, 1.335) | 1.124 (0.691, 1.826) |
| GWQS index1 | **1.382 (1.047, 1.825)** | **1.879 (1.111, 3.180)** | **1.311 (1.308, 1.314)** | 1.482 (0.868, 2.531) | 0.968 (0.397, 2.362) | **1.182 (1.179, 1.184)** |
| ***PFSAs ^b^*** |  |  |  |  |  |  |
| linear-PFHxS | 1.208 (0.945, 1.546) | 1.276 (0.680, 2.393) | 0.953 (0.647, 1.405) | 1.218 (0.800, 1.856) | 0.739 (0.460, 1.189) | 1.461 (0.872, 2.449) |
| br-PFHxS | 1.030 (0.871, 1.219) | 0.943 (0.684, 1.301) | 0.780 (0.588, 1.035) | **0.717 (0.526, 0.978)** | 0.801 (0.559, 1.149) | 1.022 (0.794, 1.315) |
| 6:2 Cl-PFESA | 1.209 (0.892, 1.639) | **1.453 (1.451, 1.455)** | 1.292 (0.771, 2.163) | 1.207 (0.707, 2.060) | 0.889 (0.523, 1.508) | 1.129 (0.623, 2.048) |
| linear-PFOS | 1.156 (0.887, 1.508) | 1.335 (0.800, 2.228) | 1.184 (0.767, 1.829) | 1.546 (0.979, 2.443) | 1.141 (0.617, 2.108) | 1.264 (0.750, 2.132) |
| br-PFOS | 1.054 (0.796, 1.397) | 1.148 (0.706, 1.865) | 1.082 (0.677, 1.729) | 1.382 (0.803, 2.377) | 1.257 (0.755, 2.094) | **1.123 (1.121, 1.126)** |
| PFHpS | 0.912 (0.793, 1.048) | 0.936 (0.655, 1.338) | 0.861 (0.689, 1.076) | 0.937 (0.746, 1.178) | 0.782 (0.609, 1.005) | 0.820 (0.660, 1.018) |
| 8:2 Cl-PFESA | 1.006 (0.835, 1.214) | 0.899 (0.654, 1.235) | 0.842 (0.632, 1.122) | 0.959 (0.689, 1.335) | 1.058 (0.564, 1.986) | 0.902 (0.663, 1.227) |
| GWQS index2 | 1.037 (0.782, 1.374) | **1.182 (1.181, 1.184)** | 1.067 (0.663, 1.716) | 1.430 (0.915, 2.233) | 0.854 (0.159, 4.576) | 1.148 (0.613, 2.153) |

*^a^* Sum of PFBA, PFHxA, PFOA, PFNA, PFDA, PFUnDA, PFDoDA, and PFTrDA.

*^b^* Sum of linear-PFHxS, br-PFHxS, 6:2 Cl-PFESA, linear-PFOS, br-PFOS, PFHpS, and 8:2 Cl-PFESA.

GWQS index 1 and GWQS index 2 were respectively created using all types of PFCAs and PFSAs to reflect their mixture exposure levels.

Odds ratios and 95% confidence intervals are reported to three decimal places due to numerically close confidence interval bounds. Values in bold represent *P*< 0.05. The models were adjusted for pre-pregnancy BMI, parity, maternal education, family income, nutrient supplementation during pregnancy, infant sex, maternal age, delivery method, birthweight, and breastfeeding duration.

Table S10. Associations of different classes of PFAS with neurodevelopment at 3 months of age (*n* = 416).

| Exposure | ASQ | Communication | Gross motor  function | Fine motor function | Problem-solving ability | Personal-social skills |
| --- | --- | --- | --- | --- | --- | --- |
|  | β (95% CI) | β (95% CI) | β (95% CI) | β (95% CI) | β (95% CI) | β (95% CI) |
| ***PFCAs*** |  |  |  |  |  |  |
| PFBA | -1.34 (-3.14, 0.45) | **-0.66 (-1.21, -0.10)** | -0.26 (-0.71, 0.19) | -0.03 (-0.51, 0.45) | 0.04 (-0.38, 0.46) | -0.44 (-0.88, 0.00) |
| PFHxA | -2.76 (-5.56, 0.05) | **-1.40 (-2.27, -0.54)** | -0.35 (-1.06, 0.36) | -0.50 (-1.24, 0.25) | -0.07 (-0.72, 0.59) | -0.44 (-1.13, 0.25) |
| PFOA | **-7.92 (-13.01, -2.83)** | **-2.64 (-4.22, -1.06)** | **-1.56 (-2.85, -0.27)** | **-1.42 (-2.78, -0.06)** | -0.98 (-2.17, 0.22) | **-1.33 (-2.58, -0.07)** |
| PFNA | -2.18 (-10.05, 5.68) | 0.48 (-1.97, 2.93) | 0.25 (-1.73, 2.23) | -1.62 (-3.71, 0.47) | -0.83 (-2.66, 1.00) | -0.46 (-2.39, 1.47) |
| PFDA | -4.38 (-11.23, 2.46) | 0.01 (-2.13, 2.14) | -0.13 (-1.86, 1.59) | **-2.09 (-3.91, -0.27)** | -1.47 (-3.06, 0.12) | -0.70 (-2.38, 0.98) |
| PFUnDA | -3.31 (-9.99, 3.37) | -0.16 (-2.24, 1.92) | -0.43 (-2.11, 1.25) | -1.24 (-3.02, 0.54) | -1.05 (-2.60, 0.51) | -0.44 (-2.08, 1.20) |
| PFDoDA | -1.12 (-4.58, 2.35) | 0.05 (-1.03, 1.13) | -0.33 (-1.21, 0.54) | -0.39 (-1.31, 0.53) | -0.17 (-0.97, 0.64) | -0.27 (-1.12, 0.58) |
| PFTrDA | 0.76 (-4.83, 6.35) | 0.33 (-1.41, 2.07) | 0.62 (-0.79, 2.02) | -0.21 (-1.70, 1.28) | -0.59 (-1.89, 0.71) | 0.61 (-0.76, 1.98) |
| ***PFSAs*** |  |  |  |  |  |  |
| linear-PFHxS | 0.66 (-3.53, 4.85) | 0.37 (-0.94, 1.67) | 0.16 (-0.90, 1.21) | -0.13 (-1.24, 0.99) | 0.22 (-0.76, 1.19) | 0.05 (-0.98, 1.08) |
| br-PFHxS | -0.06 (-3.57, 3.45) | 0.03 (-1.06, 1.12) | -0.28 (-1.16, 0.60) | 0.25 (-0.69, 1.18) | -0.13 (-0.95, 0.69) | 0.07 (-0.79, 0.93) |
| 6:2 Cl-PFESA | 2.92 (-2.75, 8.59) | 1.71 (-0.05, 3.47) | 0.34 (-1.09, 1.77) | -0.06 (-1.57, 1.46) | 0.23 (-1.09, 1.56) | 0.69 (-0.70, 2.08) |
| linear-PFOS | -0.54 (-5.48, 4.40) | 0.28 (-1.26, 1.82) | 0.21 (-1.03, 1.46) | -0.77 (-2.09, 0.54) | -0.31 (-1.46, 0.84) | 0.05 (-1.16, 1.26) |
| br-PFOS | -0.81 (-6.06, 4.44) | 0.06 (-1.58, 1.70) | -0.04 (-1.36, 1.28) | -0.60 (-2.00, 0.80) | -0.18 (-1.41, 1.04) | -0.04 (-1.33, 1.25) |
| PFHpS | -0.65 (-3.39, 2.09) | -0.10 (-0.96, 0.75) | 0.10 (-0.59, 0.79) | -0.39 (-1.12, 0.34) | -0.13 (-0.77, 0.51) | -0.13 (-0.80, 0.54) |
| 8:2 Cl-PFESA | 0.93 (-2.61, 4.46) | 0.70 (-0.39, 1.80) | -0.01 (-0.90, 0.88) | 0.39 (-0.55, 1.33) | -0.09 (-0.91, 0.74) | -0.07 (-0.93, 0.80) |

Values in bold represent *P* < 0.05. The models were adjusted for parity, maternal education, family income, nutrient supplementation during pregnancy, infant sex, birth weight, and maternal age.

Table S11. Associations of different classes of PFAS with neurodevelopment at 6 months of age (*n* = 385).

| Exposure | ASQ | Communication | Gross motor  function | Fine motor function | Problem-solving ability | Personal-social skills |
| --- | --- | --- | --- | --- | --- | --- |
|  | β (95% CI) | β (95% CI) | β (95% CI) | β (95% CI) | β (95% CI) | β (95% CI) |
| ***PFCAs*** |  |  |  |  |  |  |
| PFBA | -1.54 (-3.30, 0.23) | **-0.49 (-0.93, -0.05)** | -0.32 (-0.89, 0.24) | -0.22 (-0.69, 0.26) | -0.40 (-0.86, 0.07) | -0.11 (-0.64, 0.42) |
| PFHxA | -1.26 (-3.96, 1.43) | -1.36 (-2.03, -0.70) | -0.14 (-0.99, 0.72) | -0.15 (-0.87, 0.58) | -0.09 (-0.81, 0.62) | 0.47 (-0.33, 1.28) |
| PFOA | **-6.09 (-11.55, -0.64)** | **-2.36 (-3.73, -0.99)** | -1.18 (-2.92, 0.57) | **-1.81 (-3.27, -0.34)** | -0.39 (-1.85, 1.06) | -0.36 (-1.99, 1.28) |
| PFNA | -2.40 (-9.80, 5.00) | -1.15 (-3.02, 0.72) | -0.55 (-2.90, 1.81) | -0.93 (-2.92, 1.06) | -1.24 (-3.20, 0.72) | 1.47 (-0.72, 3.67) |
| PFDA | -3.83 (-10.13, 2.46) | -0.74 (-2.33, 0.85) | -0.98 (-2.98, 1.03) | -1.06 (-2.75, 0.63) | -1.41 (-3.08, 0.25) | 0.35 (-1.52, 2.23) |
| PFUnDA | -0.51 (-5.95, 4.94) | 0.28 (-1.10, 1.65) | -0.50 (-2.23, 1.23) | -0.26 (-1.72, 1.21) | -0.39 (-1.83, 1.06) | 0.36 (-1.26, 1.98) |
| PFDoDA | 0.03 (-3.21, 3.28) | 0.08 (-0.74, 0.90) | 0.07 (-0.96, 1.10) | 0.12 (-0.75, 0.99) | -0.44 (-1.30, 0.42) | 0.21 (-0.75, 1.18) |
| PFTrDA | 0.98 (-4.38, 6.34) | 0.36 (-1.00, 1.71) | -0.04 (-1.75, 1.66) | -0.36 (-1.80, 1.08) | 0.33 (-1.09, 1.76) | 0.69 (-0.90, 2.29) |
| ***PFSAs*** |  |  |  |  |  |  |
| linear-PFHxS | -2.63 (-6.61, 1.35) | -0.88 (-1.89, 0.12) | -0.79 (-2.06, 0.47) | -0.13 (-1.20, 0.94) | -0.69 (-1.74, 0.37) | -0.14 (-1.32, 1.05) |
| br-PFHxS | 2.74 (-0.52, 6.00) | 0.08 (-0.75, 0.90) | **1.22 (0.19, 2.25)** | 0.60 (-0.27, 1.48) | 0.64 (-0.23, 1.50) | 0.20 (-0.77, 1.17) |
| 6:2 Cl-PFESA | -0.91 (-6.28, 4.45) | -0.60 (-1.95, 0.76) | -0.54 (-2.25, 1.16) | -0.34 (-1.78, 1.10) | 0.03 (-1.39, 1.46) | 0.53 (-1.06, 2.13) |
| linear-PFOS | -0.81 (-5.73, 4.11) | -0.89 (-2.13, 0.35) | -0.32 (-1.89, 1.24) | -0.14 (-1.46, 1.18) | -0.12 (-1.43, 1.18) | 0.66 (-0.80, 2.13) |
| br-PFOS | -0.73 (-5.82, 4.36) | -0.73 (-2.02, 0.55) | -0.38 (-2.00, 1.24) | 0.06 (-1.30, 1.43) | -0.54 (-1.89, 0.81) | 0.86 (-0.65, 2.38) |
| PFHpS | -1.68 (-4.28, 0.93) | -0.63 (-1.28, 0.03) | -0.65 (-1.48, 0.17) | 0.01 (-0.69, 0.71) | -0.33 (-1.02, 0.36) | -0.08 (-0.86, 0.69) |
| 8:2 Cl-PFESA | 1.54 (-1.86, 4.95) | **0.88 (0.02, 1.73)** | 0.31 (-0.77, 1.39) | 0.12 (-0.80, 1.03) | 0.40 (-0.50, 1.31) | -0.16 (-1.18, 0.85) |

Values in bold represent *P* < 0.05. The models were adjusted for pre-pregnancy BMI, parity, maternal education, family income, nutrient supplementation during pregnancy, infant sex, alcohol consumption, and gestational age.

Table S12. Associations of different classes of PFAS with neurodevelopment at 12 months of age (*n* = 294).

| Exposure | ASQ | Communication | Gross motor  function | Fine motor function | Problem solving ability | Personal-social skills |
| --- | --- | --- | --- | --- | --- | --- |
|  | β (95% CI) | β (95% CI) | β (95% CI) | β (95% CI) | β (95% CI) | β (95% CI) |
| ***PFCAs*** |  |  |  |  |  |  |
| PFBA | -0.01 (-2.10, 2.09) | 0.18 (-0.42, 0.77) | -0.14 (-0.87, 0.59) | -0.01 (-0.47, 0.45) | 0.00 (-0.61, 0.61) | -0.03 (-0.57, 0.51) |
| PFHxA | **-4.07 (-7.27, -0.87)** | -0.65 (-1.57, 0.27) | **-1.15 (-2.27, -0.03)** | -0.53 (-1.24, 0.18) | -0.69 (-1.63, 0.25) | **-1.05 (-1.88, -0.22)** |
| PFOA | 3.32 (-2.89, 9.53) | 1.28 (-0.49, 3.05) | 1.18 (-0.99, 3.35) | 0.79 (-0.57, 2.16) | -0.21 (-2.03, 1.61) | 0.28 (-1.34, 1.90) |
| PFNA | -3.40 (-12.24, 5.44) | -0.69 (-3.21, 1.83) | -0.04 (-3.13, 3.05) | -1.14 (-3.08, 0.80) | -0.35 (-2.94, 2.23) | -1.18 (-3.47, 1.12) |
| PFDA | -2.69 (-10.54, 5.16) | -0.36 (-2.60, 1.88) | -0.59 (-3.33, 2.15) | -0.98 (-2.70, 0.75) | -0.56 (-2.85, 1.74) | -0.21 (-2.25, 1.84) |
| PFUnDA | 0.36 (-5.88, 6.61) | -0.06 (-1.85, 1.72) | -0.03 (-2.21, 2.15) | 0.30 (-1.07, 1.67) | -0.07 (-1.89, 1.75) | 0.23 (-1.40, 1.85) |
| PFDoDA | 0.36 (-3.81, 4.52) | -0.51 (-1.70, 0.67) | -0.26 (-1.71, 1.20) | 0.54 (-0.37, 1.46) | 0.39 (-0.83, 1.60) | 0.19 (-0.89, 1.28) |
| PFTrDA | 1.32 (-4.95, 7.59) | 0.61 (-1.18, 2.39) | 0.63 (-1.55, 2.82) | -0.36 (-1.74, 1.02) | 0.04 (-1.79, 1.86) | 0.41 (-1.22, 2.04) |
| ***PFSAs*** |  |  |  |  |  |  |
| linear-PFHxS | -4.61 (-9.55, 0.33) | -0.68 (-2.09, 0.74) | -0.35 (-2.08, 1.38) | -0.67 (-1.76, 0.42) | **-1.84 (-3.27, -0.40)** | -1.08 (-2.36, 0.21) |
| br-PFHxS | 2.41 (-1.51, 6.32) | 0.01 (-1.11, 1.13) | 1.20 (-0.16, 2.57) | **0.97 (0.12, 1.83)** | 0.95 (-0.19, 2.09) | -0.73 (-1.75, 0.28) |
| 6:2 Cl-PFESA | -4.33 (-10.78, 2.13) | -0.40 (-2.25, 1.45) | -1.24 (-3.49, 1.02) | -1.18 (-2.60, 0.23) | -0.89 (-2.78, 1.00) | -0.61 (-2.29, 1.07) |
| linear-PFOS | -1.39 (-6.92, 4.14) | -0.27 (-1.85, 1.31) | 0.43 (-1.50, 2.36) | -0.84 (-2.05, 0.37) | -0.36 (-1.97, 1.25) | -0.35 (-1.79, 1.09) |
| br-PFOS | -0.52 (-6.61, 5.58) | 0.05 (-1.69, 1.79) | 0.92 (-1.21, 3.04) | -0.43 (-1.76, 0.91) | -0.61 (-2.39, 1.16) | -0.44 (-2.02, 1.14) |
| PFHpS | -1.30 (-4.43, 1.83) | -0.42 (-1.31, 0.47) | -0.05 (-1.14, 1.04) | -0.10 (-0.79, 0.59) | -0.53 (-1.44, 0.39) | -0.20 (-1.02, 0.61) |
| 8:2 Cl-PFESA | -1.11 (-5.25, 3.02) | -0.45 (-1.63, 0.73) | -0.99 (-2.43, 0.45) | 0.23 (-0.68, 1.14) | -0.07 (-1.28, 1.14) | 0.17 (-0.91, 1.24) |

Values in bold represent *P* < 0.05. The models were adjusted for parity, maternal education, family income, nutrient supplementation during pregnancy, infant sex, birth weight, and maternal age.

Table S13. Associations of different classes of PFAS with neurodevelopment at 18 months of age (*n* = 259).

| Exposure | ASQ | Communication | Gross motor  function | Fine motor function | Problem-solving ability | Personal-social skills |
| --- | --- | --- | --- | --- | --- | --- |
|  | β (95% CI) | β (95% CI) | β (95% CI) | β (95% CI) | β (95% CI) | β (95% CI) |
| ***PFCAs*** |  |  |  |  |  |  |
| PFBA | 0.75 (-1.07, 2.57) | 0.28 (-0.39, 0.95) | -0.06 (-0.51, 0.38) | 0.12 (-0.41, 0.65) | 0.21 (-0.26, 0.69) | 0.19 (-0.28, 0.67) |
| PFHxA | -0.99 (-3.78, 1.80) | -0.26 (-1.28, 0.77) | -0.10 (-0.79, 0.58) | -0.34 (-1.16, 0.47) | -0.17 (-0.91, 0.56) | -0.12 (-0.85, 0.62) |
| PFOA | -6.47 (-12.99, 0.06) | -1.69 (-4.09, 0.72) | -0.50 (-2.11, 1.11) | **-2.18 (-4.08, -0.28)** | -0.51 (-2.23, 1.21) | -1.59 (-3.30, 0.12) |
| PFNA | 1.22 (-6.16, 8.60) | 0.35 (-2.36, 3.06) | 1.24 (-0.56, 3.04) | -0.22 (-2.37, 1.93) | 0.13 (-1.81, 2.07) | -0.28 (-2.21, 1.66) |
| PFDA | 0.06 (-6.73, 6.85) | 0.73 (-1.77, 3.22) | 0.78 (-0.88, 2.44) | -0.44 (-2.41, 1.54) | -0.35 (-2.13, 1.43) | -0.66 (-2.44, 1.12) |
| PFUnDA | -0.66 (-6.35, 5.03) | 0.39 (-1.70, 2.48) | 0.42 (-0.97, 1.81) | -0.73 (-2.38, 0.93) | -0.40 (-1.90, 1.09) | -0.35 (-1.84, 1.15) |
| PFDoDA | -0.93 (-4.62, 2.76) | 0.24 (-1.11, 1.60) | -0.22 (-1.13, 0.68) | -0.23 (-1.30, 0.85) | -0.13 (-1.10, 0.84) | -0.60 (-1.57, 0.36) |
| PFTrDA | 0.14 (-5.70, 5.99) | 0.84 (-1.31, 2.98) | 0.45 (-0.98, 1.87) | -0.26 (-1.97, 1.44) | -0.39 (-1.92, 1.14) | -0.49 (-2.02, 1.05) |
| ***PFSAs*** |  |  |  |  |  |  |
| linear-PFHxS | **-6.47 (-10.51, -2.43)** | **-1.87 (-3.37, -0.38)** | -0.43 (-1.44, 0.58) | **-1.24 (-2.43, -0.05)** | -1.00 (-2.07, 0.08) | **-1.93 (-2.98, -0.88)** |
| br-PFHxS | -0.88 (-4.14, 2.39) | -0.24 (-1.44, 0.96) | 0.12 (-0.68, 0.92) | 0.20 (-0.75, 1.16) | -0.31 (-1.17, 0.55) | -0.65 (-1.50, 0.20) |
| 6:2 Cl-PFESA | -2.22 (-7.79, 3.36) | -0.74 (-2.79, 1.31) | 0.26 (-1.10, 1.63) | -0.37 (-2.00, 1.26) | -0.69 (-2.15, 0.78) | -0.68 (-2.14, 0.78) |
| linear-PFOS | 0.01 (-4.65, 4.67) | -0.56 (-2.27, 1.15) | 0.48 (-0.66, 1.61) | -0.42 (-1.78, 0.93) | 0.09 (-1.13, 1.31) | 0.43 (-0.79, 1.65) |
| br-PFOS | -2.40 (-7.48, 2.67) | -1.44 (-3.30, 0.42) | 0.77 (-0.46, 2.01) | -0.53 (-2.01, 0.95) | -0.65 (-1.98, 0.68) | -0.55 (-1.88, 0.78) |
| PFHpS | **-3.41 (-6.18, -0.63)** | -0.86 (-1.88, 0.17) | -0.23 (-0.91, 0.46) | -0.72 (-1.53, 0.10) | **-0.84 (-1.57, -0.11)** | **-0.76 (-1.49, -0.04)** |
| 8:2 Cl-PFESA | -1.16 (-4.79, 2.47) | 0.56 (-0.78, 1.89) | -0.47 (-1.36, 0.42) | 0.20 (-0.86, 1.26) | -0.59 (-1.54, 0.36) | -0.86 (-1.80, 0.09) |

Values in bold represent *P* < 0.05. The models were adjusted for parity, maternal education, family income, nutrient supplementation during pregnancy, infant sex, birth weight, and maternal age.

Table S14. Associations of different classes of PFAS with neurodevelopment at 24 months of age (*n* = 238).

| Exposure | ASQ | Communication | Gross motor  function | Fine motor function | Problem-solving ability | Personal-social skills |
| --- | --- | --- | --- | --- | --- | --- |
|  | β (95% CI) | β (95% CI) | β (95% CI) | β (95% CI) | β (95% CI) | β (95% CI) |
| ***PFCAs*** |  |  |  |  |  |  |
| PFBA | -0.94 (-2.97, 1.09) | -0.46 (-1.33, 0.42) | -0.39 (-0.86, 0.07) | 0.17 (-0.27, 0.60) | -0.38 (-0.94, 0.18) | 0.12 (-0.46, 0.70) |
| PFHxA | -0.85 (-3.91, 2.21) | -0.40 (-1.73, 0.92) | -0.02 (-0.72, 0.69) | -0.04 (-0.70, 0.62) | -0.51 (-1.36, 0.33) | 0.12 (-0.75, 0.99) |
| PFOA | -3.79 (-11.23, 3.66) | -0.51 (-3.74, 2.71) | -0.20 (-1.92, 1.51) | 0.15 (-1.45, 1.74) | **-2.29 (-4.33, -0.25)** | -0.93 (-3.04, 1.19) |
| PFNA | 0.95 (-6.87, 8.77) | -0.43 (-3.81, 2.95) | 0.92 (-0.87, 2.72) | -0.13 (-1.80, 1.55) | 0.56 (-1.60, 2.72) | 0.03 (-2.19, 2.24) |
| PFDA | 1.27 (-5.80, 8.34) | -0.08 (-3.13, 2.98) | 0.53 (-1.10, 2.15) | -0.30 (-1.81, 1.22) | 0.96 (-0.99, 2.91) | 0.16 (-1.84, 2.17) |
| PFUnDA | -0.41 (-6.31, 5.48) | -0.75 (-3.29, 1.80) | 0.27 (-1.08, 1.62) | -0.35 (-1.62, 0.91) | 0.54 (-1.09, 2.16) | -0.12 (-1.79, 1.55) |
| PFDoDA | -2.62 (-6.49, 1.26) | -0.86 (-2.54, 0.81) | 0.23 (-0.66, 1.13) | **-0.86 (-1.69, -0.04)** | -0.13 (-1.20, 0.94) | -0.99 (-2.09, 0.10) |
| PFTrDA | 3.27 (-2.62, 9.16) | 0.58 (-1.97, 3.13) | 0.76 (-0.59, 2.12) | -0.00 (-1.27, 1.26) | **1.75 (0.13, 3.36)** | 0.18 (-1.49, 1.85) |
| ***PFSAs*** |  |  |  |  |  |  |
| linear-PFHxS | -4.03 (-8.63, 0.58) | **-2.07 (-4.06, -0.09)** | 0.30 (-0.77, 1.36) | 0.06 (-0.94, 1.05) | -1.25 (-2.52, 0.01) | -1.05 (-2.36, 0.25) |
| br-PFHxS | -1.82 (-5.38, 1.74) | -1.21 (-2.74, 0.32) | 0.07 (-0.75, 0.89) | -0.45 (-1.21, 0.31) | 0.25 (-0.73, 1.23) | -0.48 (-1.49, 0.53) |
| 6:2 Cl-PFESA | -0.37 (-6.36, 5.62) | -1.27 (-3.85, 1.31) | 0.64 (-0.73, 2.01) | 0.18 (-1.10, 1.46) | 0.38 (-1.27, 2.04) | -0.31 (-2.01, 1.39) |
| linear-PFOS | -0.43 (-5.52, 4.66) | -1.21 (-3.40, 0.99) | 0.16 (-1.01, 1.33) | 0.05 (-1.04, 1.14) | 0.32 (-1.08, 1.72) | 0.25 (-1.19, 1.69) |
| br-PFOS | -0.84 (-6.26, 4.58) | -0.76 (-3.10, 1.58) | 0.41 (-0.84, 1.65) | 0.29 (-0.87, 1.45) | -0.55 (-2.04, 0.94) | -0.23 (-1.77, 1.30) |
| PFHpS | 0.04 (-3.01, 3.09) | -0.51 (-1.83, 0.80) | **0.84 (0.15, 1.53)** | 0.25 (-0.41, 0.90) | -0.23 (-1.07, 0.61) | -0.31 (-1.17, 0.56) |
| 8:2 Cl-PFESA | -2.96 (-7.02, 1.09) | -1.00 (-2.75, 0.76) | -0.38 (-1.32, 0.55) | -0.35 (-1.22, 0.52) | -0.34 (-1.47, 0.78) | -0.89 (-2.03, 0.26) |

Values in bold represent *P* < 0.05. The models were adjusted for parity, maternal education, family income, nutrient supplementation during pregnancy, infant sex, birth weight, and maternal age.

Table S15. Associations of different classes of PFAS with neurodevelopment at 36 months of age (*n* = 157).

| Exposure | ASQ | Communication | Gross motor  function | Fine motor function | Problem-solving ability | Personal-social skills |
| --- | --- | --- | --- | --- | --- | --- |
|  | β (95% CI) | β (95% CI) | β (95% CI) | β (95% CI) | β (95% CI) | β (95% CI) |
| ***PFCAs*** |  |  |  |  |  |  |
| PFBA | 0.55 (-2.17, 3.27) | -0.13 (-0.91, 0.66) | 0.24 (-0.37, 0.84) | 0.49 (-0.45, 1.43) | -0.05 (-0.94, 0.83) | 0.01 (-0.70, 0.72) |
| PFHxA | 0.61 (-3.91, 5.13) | 0.31 (-0.99, 1.61) | -0.02 (-1.03, 0.99) | 0.11 (-1.45, 1.68) | 0.49 (-0.97, 1.95) | -0.28 (-1.46, 0.89) |
| PFOA | 3.38 (-4.66, 11.41) | -0.13 (-2.45, 2.19) | -0.46 (-2.25, 1.34) | 1.74 (-1.03, 4.52) | 1.25 (-1.35, 3.84) | 0.99 (-1.11, 3.08) |
| PFNA | 12.79 (-1.37, 26.96) | 2.88 (-1.22, 6.97) | -0.13 (-3.32, 3.06) | **5.84 (0.98, 10.71)** | 3.25 (-1.35, 7.85) | 0.94 (-2.78, 4.67) |
| PFDA | 1.75 (-10.47, 13.98) | 1.28 (-2.24, 4.79) | -1.16 (-3.88, 1.56) | 2.04 (-2.18, 6.26) | 0.11 (-3.84, 4.07) | -0.50 (-3.69, 2.69) |
| PFUnDA | 6.23 (-6.72, 19.17) | 1.32 (-2.41, 5.05) | -0.99 (-3.88, 1.91) | 2.77 (-1.71, 7.24) | 2.46 (-1.72, 6.64) | 0.68 (-2.71, 4.06) |
| PFDoDA | 3.65 (-1.89, 9.19) | 0.88 (-0.72, 2.47) | 0.45 (-0.79, 1.69) | 0.66 (-1.27, 2.58) | 1.59 (-0.20, 3.37) | 0.08 (-1.37, 1.53) |
| PFTrDA | 10.43 (-0.23, 21.08) | 1.27 (-1.82, 4.37) | 0.60 (-1.80, 3.01) | 3.46 (-0.23, 7.15) | **3.68 (0.24, 7.12)** | 1.39 (-1.41, 4.20) |
| ***PFSAs*** |  |  |  |  |  |  |
| linear-PFHxS | 5.82 (-1.06, 12.70) | 1.20 (-0.79, 3.19) | 0.55 (-0.99, 2.10) | 1.47 (-0.92, 3.86) | **2.24 (0.02, 4.46)** | 0.36 (-1.45, 2.17) |
| br-PFHxS | 0.85 (-4.17, 5.88) | -0.24 (-1.69, 1.21) | 0.11 (-1.02, 1.23) | 1.02 (-0.71, 2.75) | 0.57 (-1.05, 2.19) | -0.60 (-1.91, 0.71) |
| 6:2 Cl-PFESA | 6.61 (-2.40, 15.63) | 0.90 (-1.71, 3.51) | 0.80 (-1.22, 2.82) | 0.89 (-2.25, 4.02) | **3.95 (1.09, 6.82)** | 0.10 (-2.26, 2.47) |
| linear-PFOS | 5.95 (-3.40, 15.31) | 0.76 (-1.94, 3.47) | -0.42 (-2.51, 1.68) | **3.85 (0.65, 7.04)** | 1.88 (-1.15, 4.91) | -0.12 (-2.57, 2.33) |
| br-PFOS | 5.35 (-3.29, 13.99) | 0.20 (-2.30, 2.70) | -0.43 (-2.37, 1.51) | **3.53 (0.58, 6.49)** | 2.19 (-0.60, 4.97) | -0.12 (-2.38, 2.14) |
| PFHpS | **6.06 (1.85, 10.28)** | **1.59 (0.38, 2.81)** | 0.38 (-0.59, 1.34) | **1.72 (0.25, 3.19)** | **1.63 (0.26, 3.00)** | 0.74 (-0.38, 1.86) |
| 8:2 Cl-PFESA | 3.01 (-2.16, 8.18) | 0.07 (-1.42, 1.57) | 0.79 (-0.36, 1.94) | -0.08 (-1.88, 1.72) | **2.12 (0.47, 3.76)** | 0.13 (-1.23, 1.48) |

Values in bold represent *P* < 0.05. The models were adjusted for parity, maternal education, family income, nutrient supplementation during pregnancy, infant sex, birth weight and maternal age.

Table S16. Associations of different classes of PFAS with neurodevelopment at 48 months of age (*n* = 92).

| Exposure | ASQ | Communication | Gross motor  function | Fine motor function | Problem-solving ability | Personal-social skills |
| --- | --- | --- | --- | --- | --- | --- |
|  | β (95% CI) | β (95% CI) | β (95% CI) | β (95% CI) | β (95% CI) | β (95% CI) |
| ***PFCAs*** |  |  |  |  |  |  |
| PFBA | 1.64 (-1.01, 4.30) | 0.26 (-0.62, 1.14) | **0.86 (0.11, 1.62)** | -0.10 (-1.04, 0.84) | 0.44 (-0.45, 1.32) | 0.18 (-0.37, 0.74) |
| PFHxA | -1.33 (-5.12, 2.47) | -0.84 (-2.08, 0.41) | 0.24 (-0.87, 1.35) | -0.70 (-2.02, 0.63) | -0.16 (-1.43, 1.11) | 0.14 (-0.65, 0.93) |
| PFOA | 7.93 (-1.71, 17.57) | 1.53 (-1.69, 4.74) | 2.33 (-0.48, 5.15) | -0.68 (-4.11, 2.75) | 1.96 (-1.28, 5.19) | **2.79 (0.85, 4.73)** |
| PFNA | -1.35 (-15.33, 12.63) | -1.29 (-5.90, 3.32) | 0.43 (-3.66, 4.51) | -1.22 (-6.12, 3.67) | -0.69 (-5.35, 3.98) | 1.42 (-1.46, 4.31) |
| PFDA | -6.34 (-18.18, 5.51) | **-4.01 (-7.85, -0.17)** | 0.22 (-3.26, 3.70) | -2.33 (-6.48, 1.82) | -1.43 (-5.39, 2.54) | 1.21 (-1.25, 3.68) |
| PFUnDA | -0.42 (-8.74, 7.89) | -0.91 (-3.65, 1.83) | -0.52 (-2.95, 1.90) | 0.61 (-2.30, 3.52) | -0.11 (-2.89, 2.66) | 0.51 (-1.21, 2.24) |
| PFDoDA | 0.31 (-4.75, 5.37) | -0.41 (-2.08, 1.26) | -0.16 (-1.64, 1.31) | 0.20 (-1.57, 1.97) | 0.52 (-1.17, 2.20) | 0.16 (-0.89, 1.21) |
| PFTrDA | 4.17 (-6.09, 14.43) | 0.79 (-2.61, 4.18) | -1.00 (-4.00, 2.00) | 1.75 (-1.84, 5.34) | 1.09 (-2.34, 4.52) | 1.54 (-0.58, 3.65) |
| ***PFSAs*** |  |  |  |  |  |  |
| linear-PFHxS | 0.57 (-4.98, 6.12) | -0.07 (-1.90, 1.76) | -0.59 (-2.21, 1.02) | 0.10 (-1.85, 2.04) | 1.01 (-0.83, 2.85) | 0.13 (-1.02, 1.28) |
| br-PFHxS | 1.80 (-2.65, 6.24) | 0.69 (-0.78, 2.16) | -0.73 (-2.02, 0.57) | 0.54 (-1.02, 2.10) | 1.08 (-0.39, 2.55) | 0.21 (-0.72, 1.14) |
| 6:2 Cl-PFESA | -3.70 (-13.54, 6.14) | -2.41 (-5.62, 0.81) | -1.22 (-4.09, 1.65) | -0.85 (-4.30, 2.61) | 0.81 (-2.48, 4.10) | -0.04 (-2.09, 2.01) |
| linear-PFOS | -1.94 (-10.65, 6.77) | -0.31 (-3.19, 2.56) | -1.90 (-4.41, 0.61) | -1.31 (-4.35, 1.73) | 0.52 (-2.38, 3.43) | 1.07 (-0.73, 2.86) |
| br-PFOS | 1.88 (-6.94, 10.69) | 0.54 (-2.37, 3.45) | 0.38 (-2.19, 2.96) | -1.35 (-4.43, 1.73) | 1.46 (-1.46, 4.39) | 0.84 (-0.98, 2.67) |
| PFHpS | -0.27 (-4.46, 3.92) | 0.55 (-0.83, 1.93) | -0.76 (-1.97, 0.45) | -0.49 (-1.95, 0.98) | 0.28 (-1.11, 1.68) | 0.15 (-0.72, 1.02) |
| 8:2 Cl-PFESA | -3.63 (-9.68, 2.42) | -1.56 (-3.55, 0.43) | -0.79 (-2.57, 0.98) | -0.73 (-2.86, 1.40) | -0.20 (-2.23, 1.84) | -0.35 (-1.62, 0.91) |

Values in bold represent *P* < 0.05. The models were adjusted for parity, maternal education, family income, nutrient supplementation during pregnancy, infant sex, birth weight, and maternal age.

Table S17. Associations of different classes of PFAS with neurodevelopment at 60 months of age (*n* = 142).

| Exposure | ASQ | Communication | Gross motor  function | Fine motor function | Problem-solving ability | Personal-social skills |
| --- | --- | --- | --- | --- | --- | --- |
|  | β (95% CI) | β (95% CI) | β (95% CI) | β (95% CI) | β (95% CI) | β (95% CI) |
| ***PFCAs*** |  |  |  |  |  |  |
| PFBA | -0.09 (-1.66, 1.47) | 0.09 (-0.49, 0.66) | -0.05 (-0.36, 0.25) | 0.06 (-0.55, 0.67) | -0.07 (-0.60, 0.47) | -0.12 (-0.41, 0.18) |
| PFHxA | -0.73 (-3.29, 1.83) | -0.22 (-1.17, 0.72) | -0.06 (-0.56, 0.43) | -0.27 (-1.27, 0.72) | -0.13 (-1.01, 0.75) | -0.05 (-0.53, 0.44) |
| PFOA | 2.28 (-3.72, 8.29) | 0.43 (-1.78, 2.65) | 0.36 (-0.80, 1.52) | 0.20 (-2.14, 2.55) | -0.03 (-2.09, 2.03) | **1.32 (0.21, 2.43)** |
| PFNA | 1.55 (-4.35, 7.45) | 0.37 (-1.81, 2.54) | -0.28 (-1.42, 0.86) | -0.25 (-2.55, 2.05) | 1.26 (-0.75, 3.28) | 0.45 (-0.67, 1.56) |
| PFDA | 0.96 (-5.00, 6.92) | 0.64 (-1.55, 2.84) | -0.32 (-1.47, 0.83) | 0.09 (-2.23, 2.42) | 0.33 (-1.71, 2.38) | 0.21 (-0.92, 1.33) |
| PFUnDA | 0.29 (-3.57, 4.15) | -0.58 (-2.00, 0.84) | -0.22 (-0.97, 0.52) | 0.35 (-1.16, 1.85) | 0.64 (-0.68, 1.96) | 0.11 (-0.62, 0.84) |
| PFDoDA | 2.07 (-1.40, 5.53) | 0.61 (-0.67, 1.89) | 0.02 (-0.65, 0.69) | 0.62 (-0.74, 1.97) | 0.37 (-0.82, 1.57) | 0.45 (-0.21, 1.10) |
| PFTrDA | 5.14 (-0.40, 10.68) | 0.81 (-1.26, 2.87) | 0.13 (-0.95, 1.21) | 1.27 (-0.91, 3.44) | **2.71 (0.84, 4.57)** | 0.23 (-0.83, 1.29) |
| ***PFSAs*** |  |  |  |  |  |  |
| linear-PFHxS | **5.86 (1.14, 10.58)** | 1.20 (-0.56, 2.97) | -0.21 (-1.15, 0.72) | 1.85 (-0.01, 3.70) | 1.55 (-0.09, 3.18) | **1.48 (0.60, 2.36)** |
| br-PFHxS | 0.74 (-2.88, 4.37) | 1.27 (-0.05, 2.59) | -0.04 (-0.74, 0.66) | -0.19 (-1.60, 1.23) | -0.33 (-1.57, 0.91) | 0.02 (-0.66, 0.71) |
| 6:2 Cl-PFESA | 3.89 (-0.49, 8.26) | 0.61 (-1.01, 2.24) | 0.15 (-0.71, 1.00) | 0.90 (-0.82, 2.62) | 1.19 (-0.31, 2.70) | **1.03 (0.22, 1.85)** |
| linear-PFOS | 1.67 (-2.18, 5.51) | -0.25 (-1.67, 1.18) | -0.00 (-0.75, 0.74) | 0.86 (-0.64, 2.36) | 0.63 (-0.69, 1.95) | 0.42 (-0.30, 1.15) |
| br-PFOS | 1.78 (-2.46, 6.02) | -0.01 (-1.58, 1.55) | -0.09 (-0.91, 0.73) | 0.62 (-1.03, 2.28) | 0.51 (-0.94, 1.97) | 0.75 (-0.04, 1.54) |
| PFHpS | **3.31 (0.39, 6.23)** | 0.56 (-0.54, 1.65) | -0.06 (-0.64, 0.51) | **1.55 (0.42, 2.68)** | 0.03 (-0.99, 1.05) | 1.24 (0.72, 1.76) |
| 8:2 Cl-PFESA | 1.49 (-1.57, 4.55) | 0.46 (-0.67, 1.59) | 0.23 (-0.37, 0.82) | 1.10 (-0.09, 2.28) | -0.90 (-1.94, 0.14) | **0.62 (0.04, 1.19)** |

Values in bold represent *P* < 0.05. The models were adjusted for parity, maternal education, family income, nutrient supplementation during pregnancy, infant sex, birth weight, and maternal age.

**Table S18.** Adjusted ORs (95% CIs) per unit increase in individual PFAS levels for developmental delay in children aged 3–60 months: A longitudinal analysis (using IPW).

| Serum PFAS (ng/mL) | ASQ | Communication | Gross motor  function | Fine motor  function | Problem-solving  ability | Personal-social skills |
| --- | --- | --- | --- | --- | --- | --- |
|  | Adjusted ORs (95% CIs) | | | | | |
| ***PFCAs*** |  |  |  |  |  |  |
| PFBA | 1.024 (0.935, 1.122) | **1.173 (1.171, 1.174)** | 1.040 (0.850, 1.273) | 0.958 (0.736, 1.247) | 0.945 (0.709, 1.260) | 0.869 (0.716, 1.055) |
| PFHxA | 0.980 (0.850, 1.130) | 1.079 (0.839, 1.388) | 0.887 (0.640, 1.229) | 0.768 (0.581, 1.015) | 0.827 (0.570, 1.200) | 0.928 (0.615, 1.399) |
| PFOA | **1.396 (1.065, 1.832)** | 1.099 (0.597, 2.023) | 0.744 (0.413, 1.338) | **1.591 (1.589, 1.593)** | 0.883 (0.511, 1.523) | 0.896 (0.492, 1.633) |
| PFNA | 1.294 (0.855, 1.957) | 1.205 (0.473, 3.074) | 0.834 (0.330, 2.110) | 2.075 (0.872, 4.939) | 0.615 (0.265, 1.425) | 0.971 (0.336, 2.801) |
| PFDA | 1.383 (0.973, 1.965) | 0.920(0.487, 1.737) | 1.088 (0.497, 2.383) | 1.802 (0.962, 3.374) | 1.747 (0.841, 3.627) | 0.965 (0.347, 2.684) |
| PFUnDA | 1.346 (0.969, 1.871) | 1.315 (0.622, 2.777) | 1.057 (0.475, 2.350) | **2.767 (1.189, 6.441)** | 1.058 (0.560, 1.998) | 1.996 (0.624, 6.379) |
| PFDoDA | 0.848 (0.666, 1.080) | 1.076 (0.628, 1.846) | 0.771 (0.406, 1.466) | 0.781 (0.443, 1.375) | 0.715 (0.405, 1.261) | 0.788 (0.423, 1.468) |
| PFTrDA | 0.957 (0.726, 1.261) | 0.627 (0.380, 1.037) | 1.003 (0.501, 2.011) | 0.881 (0.539, 1.440) | 0.764 (0.383, 1.526) | 0.826 (0.409, 1.668) |
| ***PFSAs*** |  |  |  |  |  |  |
| linear-PFHxS | 1.222 (0.970, 1.539) | 1.291 (0.854, 1.952) | 0.935 (0.566, 1.545) | 0.679 (0.399, 1.154) | 0.931 (0.602, 1.440) | 1.443 (0.881, 2.364) |
| br-PFHxS | 1.057 (0.891, 1.253) | 0.671 (0.424, 1.062) | **0.685 (0.472, 0.993)** | **0.698 (0.491, 0.993)** | **2.087 (1.274, 3.417)** | 1.126 (0.845, 1.499) |
| 6:2 Cl-PFESA | 1.259 (0.937, 1.693) | 1.047 (0.586, 1.870) | 1.535 (0.785, 3.000) | 1.108 (0.525, 2.337) | 0.713 (0.369, 1.377) | 2.159 (0.912, 5.111) |
| linear-PFOS | 1.198 (0.920, 1.560) | **1.824 (1.042, 3.195)** | 1.223 (0.763, 1.959) | 1.416 (0.851, 2.355) | 0.992 (0.435, 2.260) | 1.405 (0.840, 2.351) |
| br-PFOS | 1.171 (0.886, 1.547) | 1.278 (0.753, 2.169) | 0.741 (0.413, 1.328) | 0.816 (0.486, 1.371) | 1.564 (0.667, 3.668) | 1.663 (0.848, 3.262) |
| PFHpS | 0.923 (0.804, 1.060) | 0.833 (0.624, 1.111) | 0.875 (0.688, 1.112) | 0.836 (0.653, 1.070) | 0.913 (0.626, 1.332) | **0.750 (0.569, 0.989)** |
| 8:2 Cl-PFESA | 1.054 (0.879, 1.263) | 1.057 (0.668, 1.673) | 1.178 (0.746, 1.860) | 0.959 (0.582, 1.581) | 0.768 (0.465, 1.269) | 0.799 (0.572, 1.115) |

Odds ratios and 95% confidence intervals are reported to three decimal places due to numerically close confidence interval bounds. Values in bold represent *P* < 0.05. The models were adjusted for pre-pregnancy BMI, parity, maternal education, family income, nutrient supplementation during pregnancy, infant sex, and maternal age.


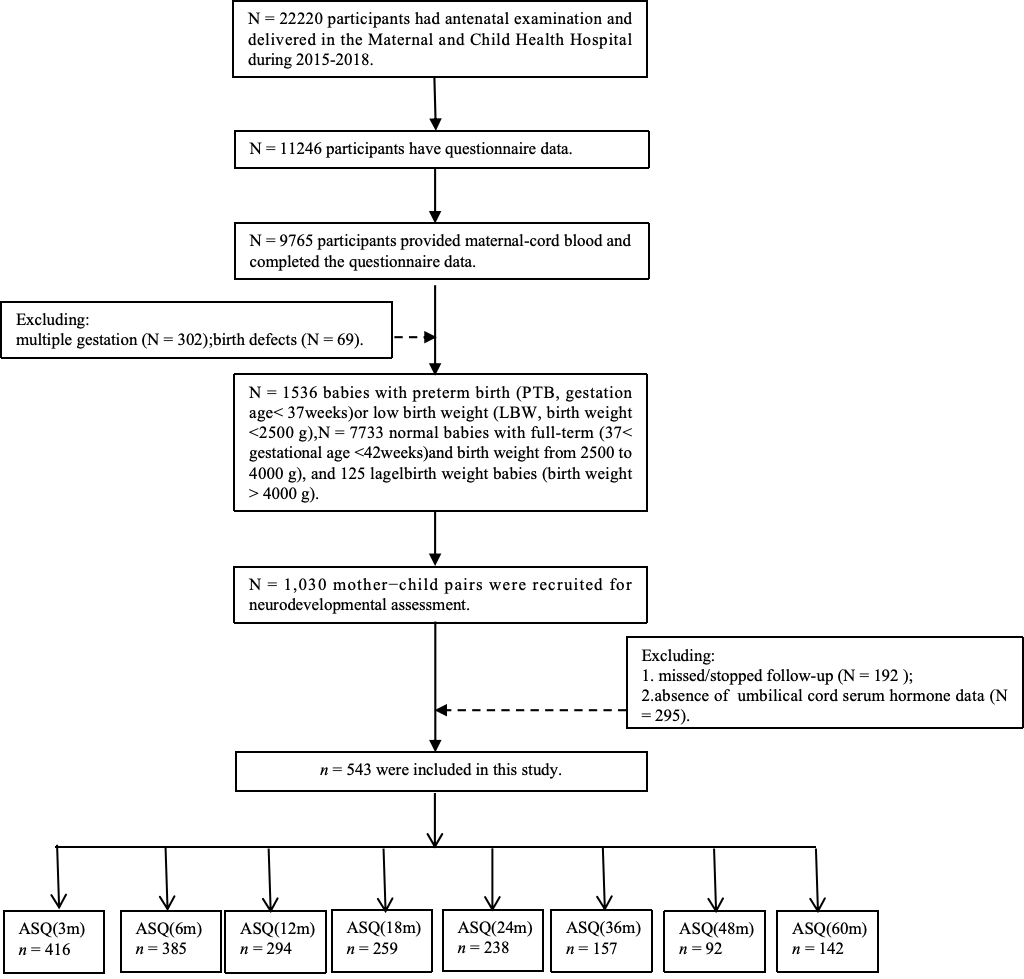


Fig. S1. Flow diagram of subjects in this study at recruitment and follow-up (*n* = 543).


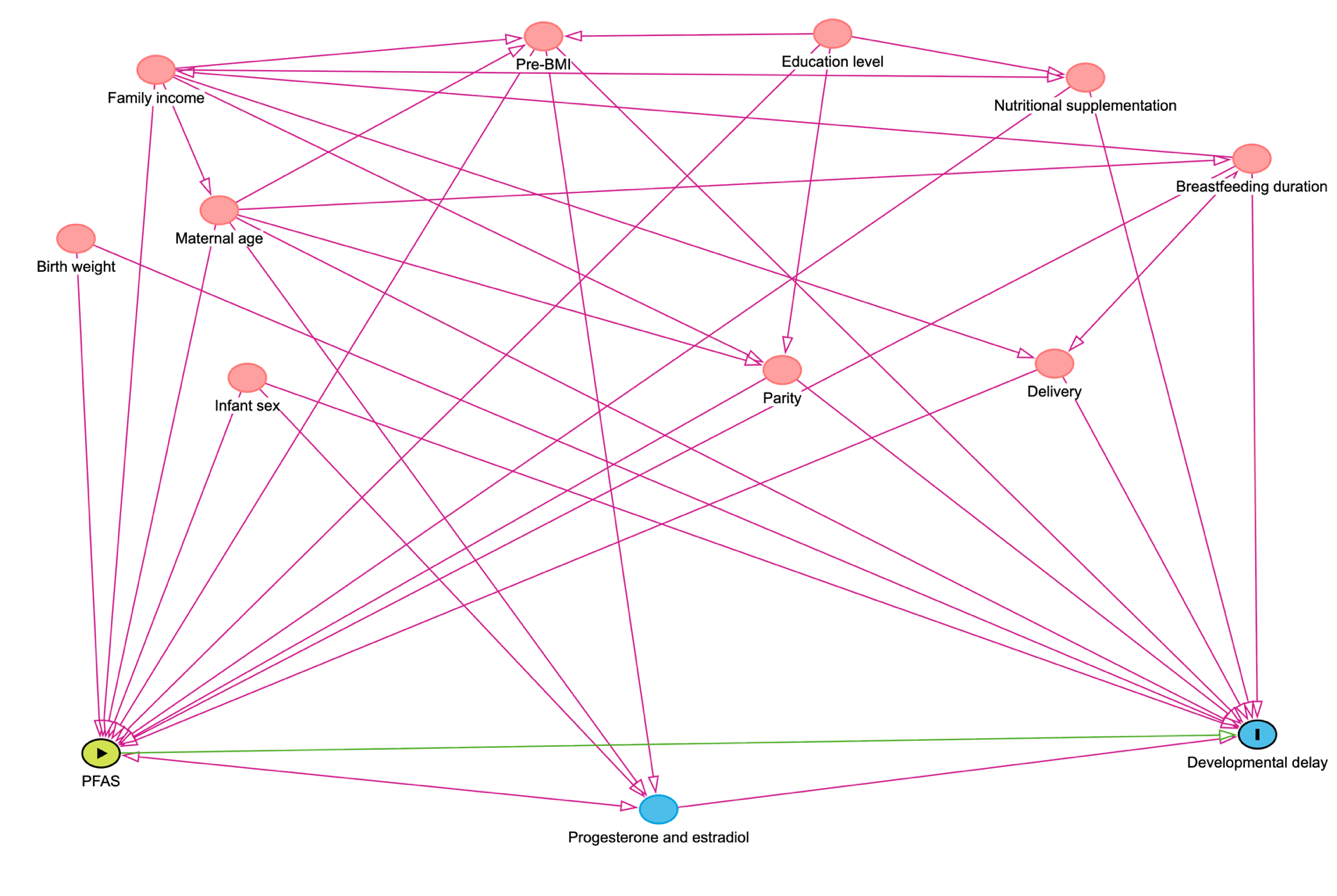


Fig. S2. Directed acyclic graph for the association between PFAS exposure and neurodevelopmental delay in Children using DAGitty (http://www.dagitty.net/). Note: green indicates ancestor of exposure; blue indicates ancestor of outcome; red indicates ancestor of both exposure and outcome.


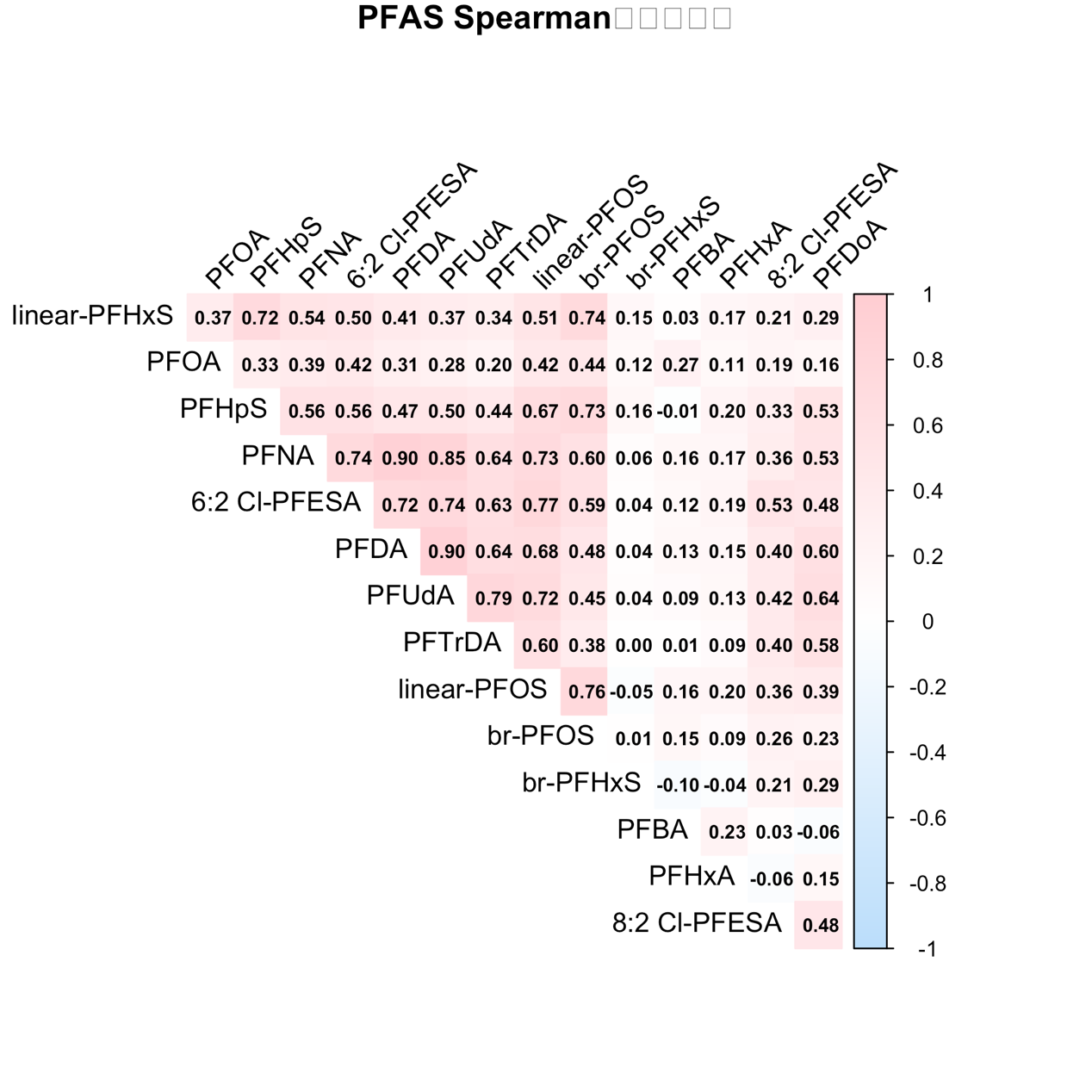


Fig. S3. The correlation plot of maternal serum PFAS levels.

**
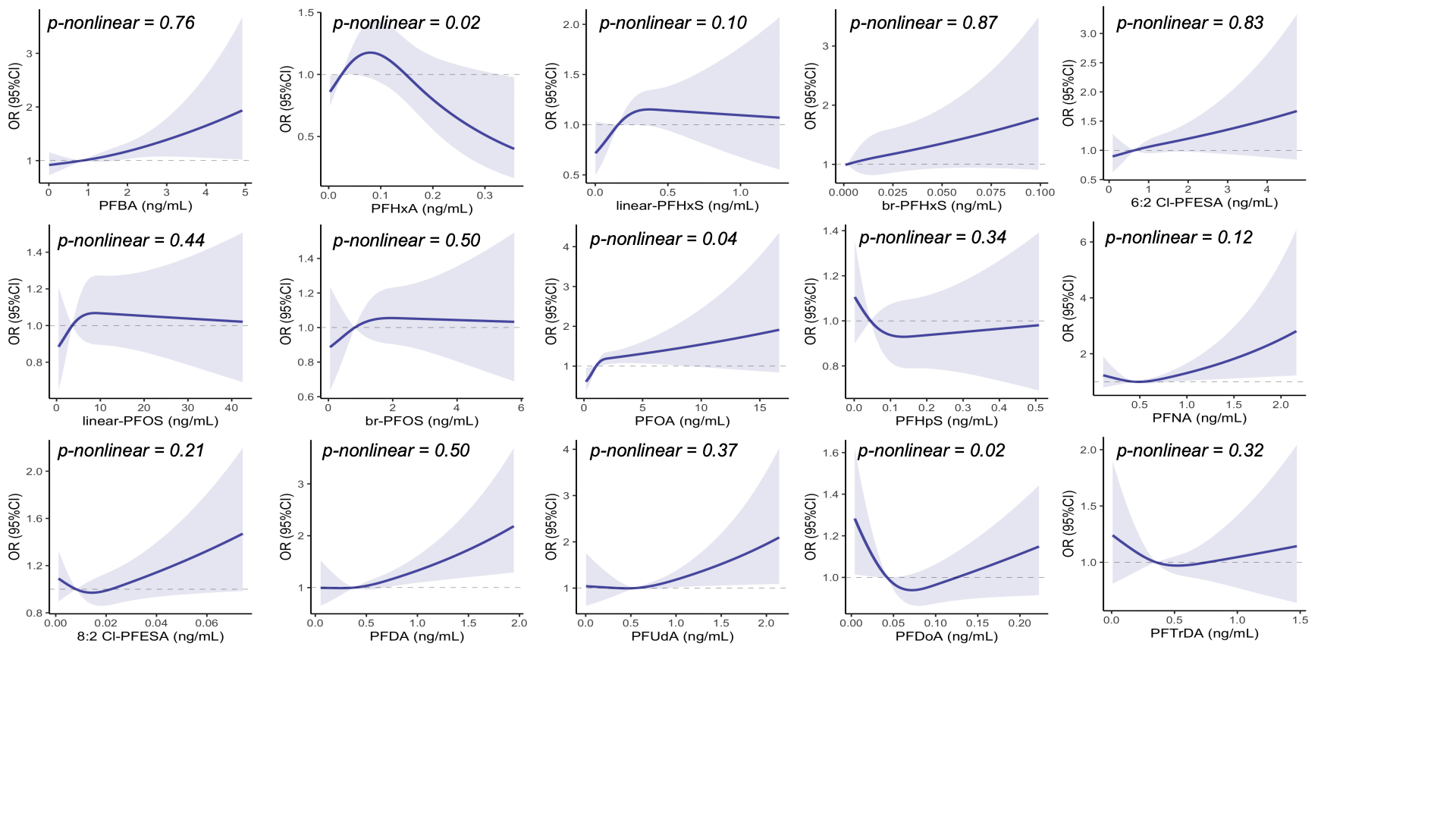
**

Fig. S4. The non-linear relationship between PFAS and ASQ (*n* = 543). The models were adjusted for parity, maternal education, family income, nutrient supplementation during pregnancy, infant sex, birth weight, and maternal age. Note: *p*-nonlinear was calculated by the restricted cubic spline model.


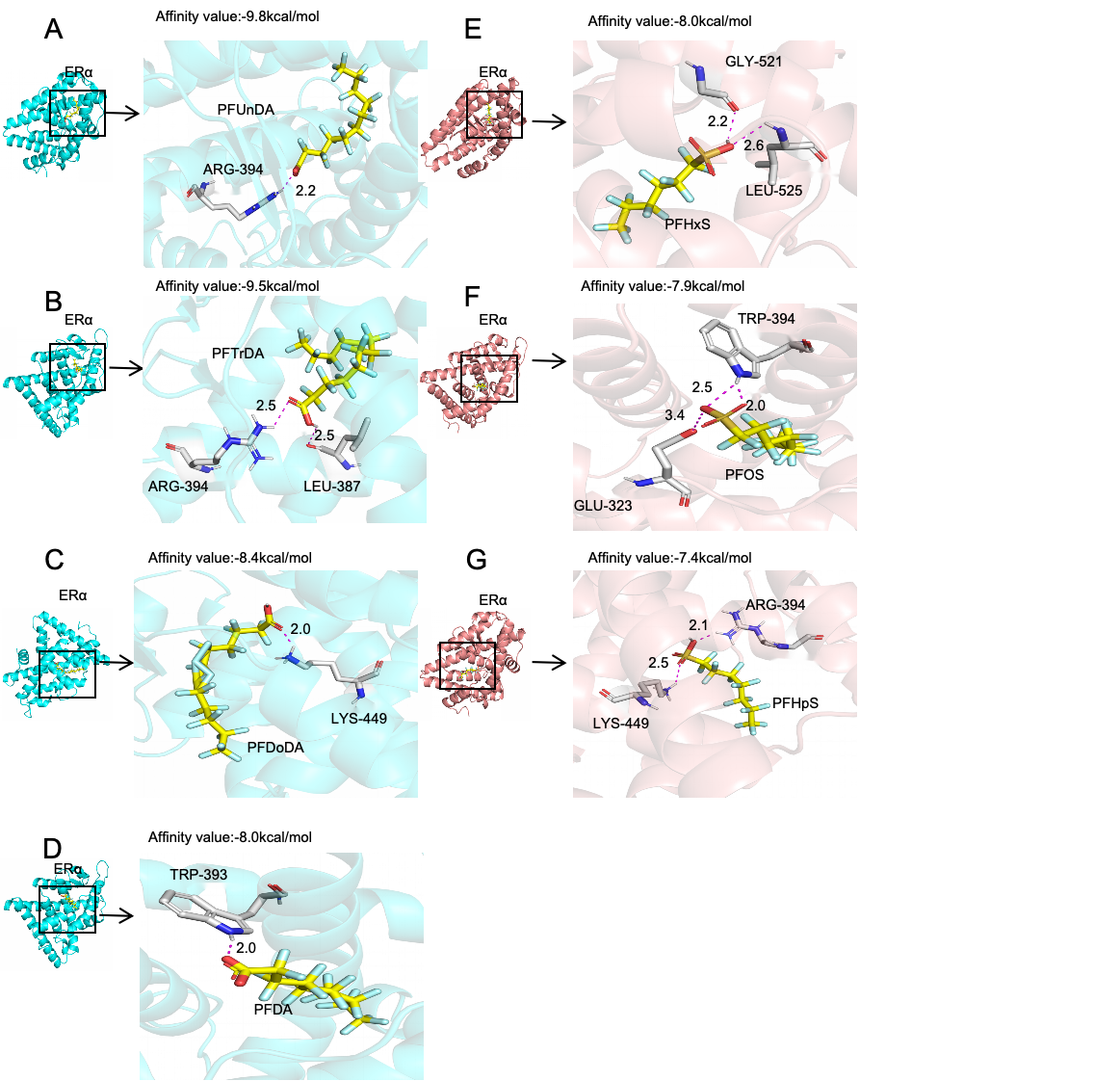


Fig. S5. Effects of PFAS compounds on the binding conformations and affinities with estrogen receptor α (ERα). Binding configurations of PFUnDA (A), PFTrDA (B), PFDoDA (C), PFDA (D), PFHxS (E), PFOS (F), and PFHpS (G) with ERα. Affinity values (kcal/mol) are shown for each complex. Blue indicates PFCAs (A–D), and red indicates PFSAs (E–G).


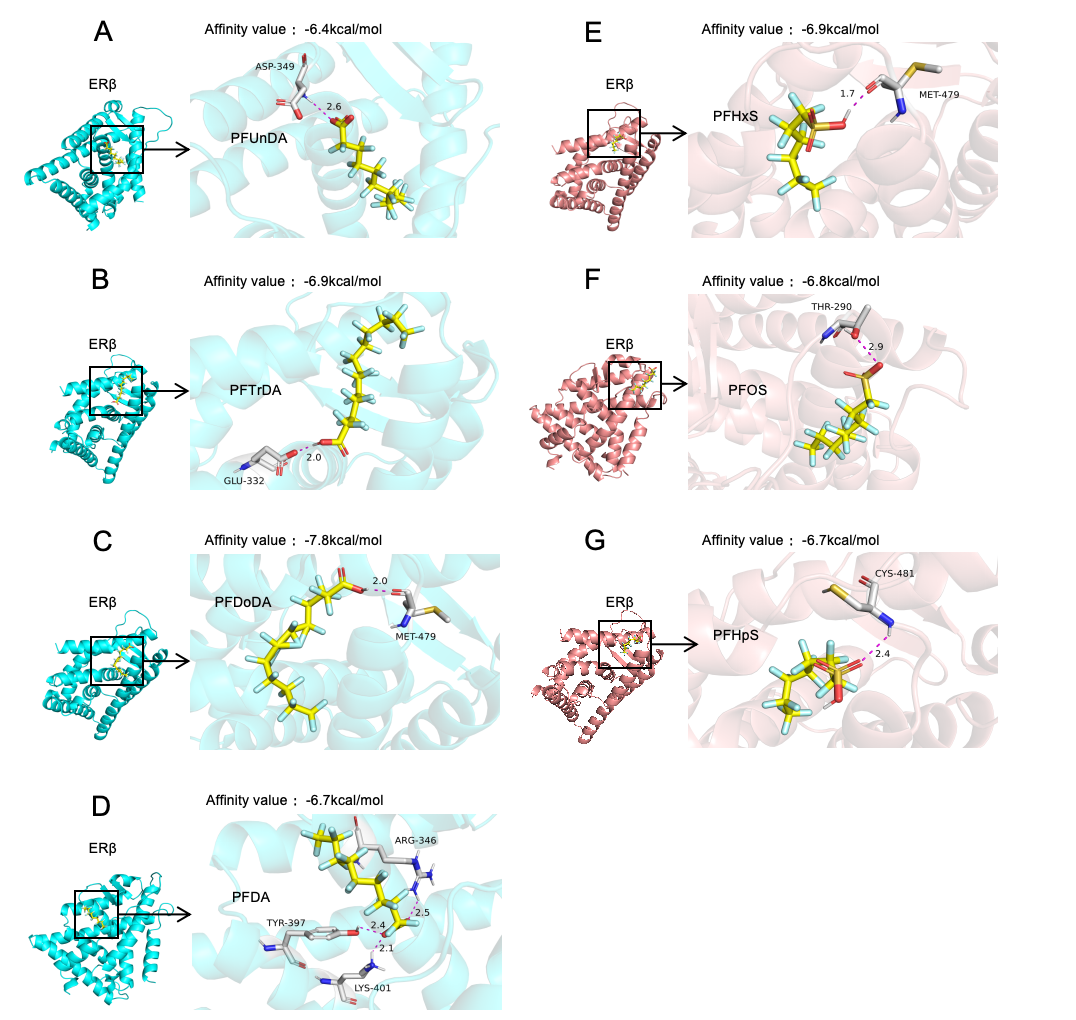


Fig. S6. Effects of PFAS compounds on the binding conformations and affinities with estrogen receptor β (ERβ). Binding configurations of PFUnDA (A), PFTrDA (B), PFDoDA (C), PFDA (D), PFHxS (E), PFOS (F), and PFHpS (G) with ERβ. Affinity values (kcal/mol) are shown for each complex. Blue indicates PFCAs (A–D), and red indicates PFSAs (E–G).


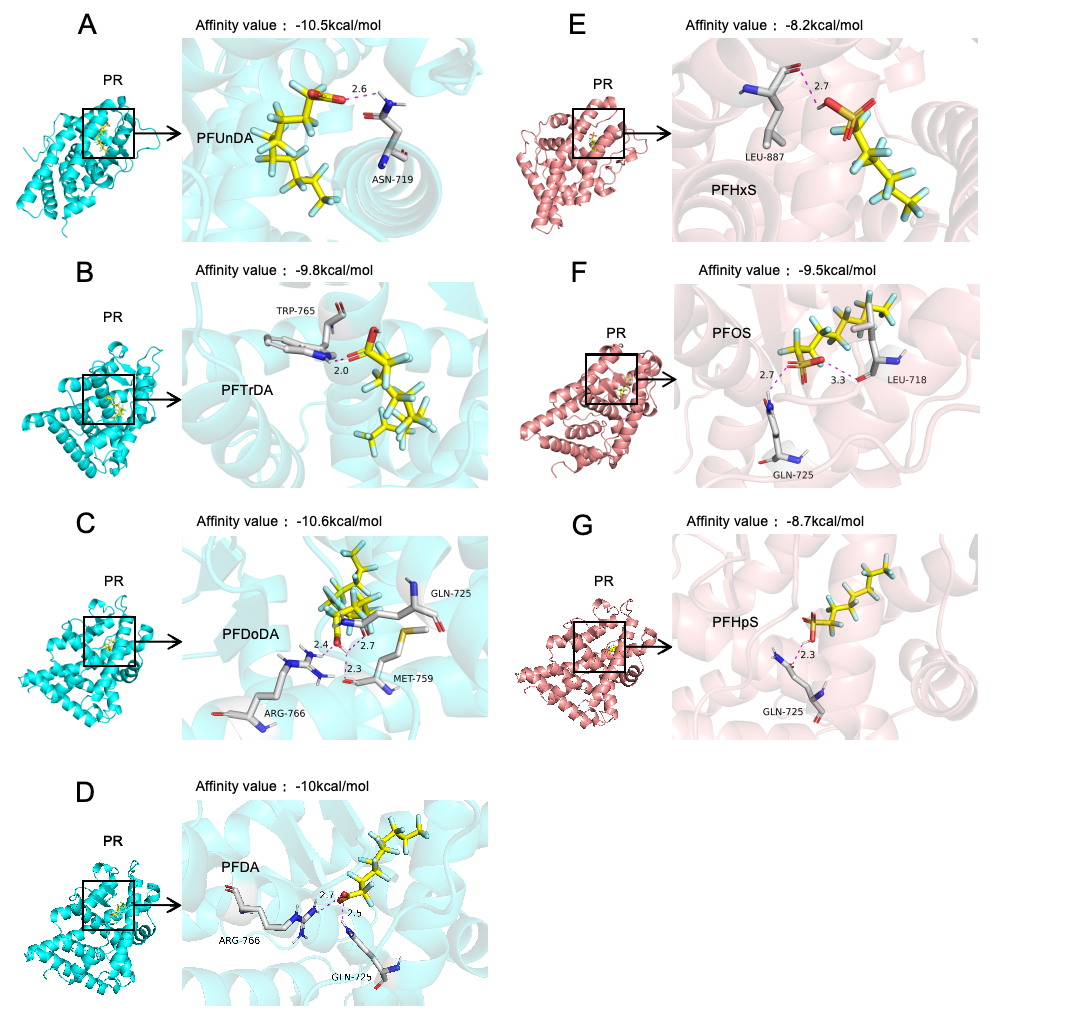


Fig. S7. Effects of PFAS compounds on the binding conformations and affinities with progesterone receptor (PR). Binding configurations of PFUnDA (A), PFTrDA (B), PFDoDA (C), PFDA (D), PFHxS (E), PFOS (F), and PFHpS (G) with PR. Affinity values (kcal/mol) are shown for each complex. Blue indicates PFCAs (A–D), and red indicates PFSAs (E–G).
